# The curse of dimensionality: Animal-related risk factors for pediatric diarrhea in western Kenya, and methods for dealing with a large number of predictors

**DOI:** 10.1371/journal.pone.0215982

**Published:** 2019-04-26

**Authors:** Julianne Meisner, Stephen J. Mooney, Peter M. Rabinowitz

**Affiliations:** 1 Department of Epidemiology, University of Washington, Seattle, WA, United States of America; 2 Center for One Health Research, University of Washington, Seattle, WA, United States of America; 3 Harborview Injury & Prevention Research Center, University of Washington, Seattle, WA, United States of America; 4 Department of Environmental and Occupational Health Sciences, University of Washington, Seattle, WA, United States of America; 5 Department of Global Health, University of Washington, Seattle, WA, United States of America; Johns Hopkins Bloomberg School of Public Health, UNITED STATES

## Abstract

**Background:**

Pediatric diarrhea, a leading cause of under-five mortality, is predominantly infectious in etiology. As many putative causal agents are zoonotic, animal exposure is a likely risk factor. To evaluate the effect of animal-related factors on moderate to severe childhood diarrhea in rural Kenya, where animal contact is common, Conan *et al*. studied 73 matched case-control pairs from 2009-2011, collecting rich exposure data on many dimensions of animal contact. We review the challenges associated with analyzing moderately-sized datasets with a large number of predictors and present two alternative methodological approaches.

**Methodology/Principal findings:**

We conducted a simulation study to demonstrate that forward stepwise selection results in overfit models when data are high-dimensional, and that p values reported directly from the data used to train these models are misleading. We described how automated methods of variable selection, attractive when the number of predictors is large, can result in overadjustment bias. We proposed an alternative *a priori* regression approach not subject to this bias. Applied to Conan *et al*.’s data, this approach found a non-significant but positive trend for household’s sharing of water sources with livestock or poultry, child’s presence for poultry slaughter, and child’s habit of playing where poultry sleep or defecate. For many predictors evaluated few pairs were discordant, suggesting matching compromised the power of this analysis. Finally, we proposed latent variable modeling as a complimentary approach and performed Item Response Theory modeling on Conan *et al*.’*s* data, with animal contact as the latent trait. We found a moderate but non-significant effect (OR 1.21, 95% CI 0.78, 1.87, unit = 1 standard deviation).

**Conclusions/Significance:**

Automated methods of model selection are appropriate for prediction models when fit and evaluated on separate samples. However when the goal is inference, these methods can produce misleading results. Furthermore, case-control matching should be done with caution.

## Introduction

Diarrheal disease is the leading cause of pediatric malnutrition and the second leading cause of under five mortality, with over 1.7 billion childhood cases and more than 500,000 under-five deaths each year [1]. Pediatric diarrhea is predominantly caused by bacterial, viral, and parasitic infections, and animals are known reservoirs of several important diarrheal pathogens, including *Campylobacter* spp., *Salmonella* spp., and *Escherichia coli* [2]. In low-resource settings, where animal keeping is a predominant source of income, animal contact is common in both pediatric and adult populations.

The Global Enteric Multicenter Study (GEMS) is a large-scale case-control study of pediatric diarrhea conducted across four sites in sub-Saharan African and three sites in south Asia from January 2008-2011. The methodology and findings of GEMS have been published elsewhere [3–6]. To identify animal-related risk factors for moderate-to-severe diarrhea, the GEMS Zoonotic Enteric Diseases (GEMS-ZED) sub-study was conducted from November 2009-February 2011 among subjects enrolled at one of the six GEMS sites in rural western Kenya. This study, whose methods and findings are published PLOS NTD [7] and summarized here, represented an impressive effort to collect detailed data on animals and animal-related exposures in a matched case-control pediatric population, thereby addressing an important scientific question: to what extent does direct and indirect contact with animals and animal excreta bear on pediatric diarrheal disease?

The GEMS-ZED substudy was conducted on 73 case-control pairs with reported animal presence at home, including domestic animals and peridomestic wild rodents. Cases were matched to their controls on age (± 2 months for cases 0-11 months, ±4 months for cases 12-59 months, not exceeding stratum boundaries for the case), sex, and location (lives in the same or nearby village/neighborhood as the case) [3]. The head of the compound and the child’s caregiver were interviewed using a standard questionnaire, which collected data on presence and husbandry of domestic animals, observed presence of rodents in the compound, and animal exposure data specific to the participating child. This questionnaire is provided as a supplemental file in the original publication. Fecal specimens were also collected and analyzed from domestic animals belonging to the child’s compound, however the data contained in the questionnaire is the focus of this analysis.

Including recoded variables, Conan *et al*. analyzed data on 497 predictors (P). This large number of predictors presents analytic challenges, particularly given the moderate size of this dataset (N = 146). However this high dimensionality also provides the opportunity to address a large number of scientific questions, an unarguable benefit in this vulnerable population. As studies of this dimensionality (high P:N ratio) are increasingly common in One Health research, we will discuss their challenges and opportunities, and propose methodological approaches that we believe strike an appropriate balance. We will also discuss issues that can arise as a result of matching. Due to the richness of its data and the importance of Conan *et al*.’s scientific questions, the GEMS-ZED study is an excellent example to present these methods.

## Materials and methods

R version 3.4.4 was used for all analyses [8].

### Challenges with a high P:N ratio

Multivariable regression is the mainstay of epidemiologic analyses, however the selection of variables for inclusion in a model is particularly challenging when the number of candidate variables is very large. Automated approaches—including univariable screening based on p values, stepwise selection based on changes in effect estimates or model fit criteria, and numerous other methods—are attractive for their ease of implementation, increasing familiarity among readers, and reproducibility. However, these approaches carry several risks: overfitting, misleading inference if validation is not performed, and overadjustment. Furthermore, data of this dimensionality are harder hit by losses in statistical power due to overmatching.

#### Overfitting

Overfitting arises when, heuristically, “too much” is asked of the data. Under a frequentist approach, inference is performed by considering a thought experiment in which the study in question is repeated many times in the super-population from which the study population is assumed to be a random sample. When a model is overfit, small differences from one hypothetical study to the next results in large differences in resulting inference; that is, the model follows the noise in the dataset too closely [9]. When automated model selection is performed, this can result in different variables being retained in each multivariable model. Within increasingly flexible models, the risk of overfitting also increases.

While recent simulation studies suggest the traditional rule of thumb (N:P ≥ 10:1) is overly conservative, effect estimates may be strongly biased (over- or underestimated by up to a factor of 10) with N:P of 4:1 or smaller [10, 11]. Furthermore, if the exposure variability in the data is small, overfitting may occur at higher N:P ratios. This a risk in matched studies if matching variables are strongly associated with exposure.

#### Misleading inference if validation is not performed

Automated approaches to variable selection based on model fit criteria, including AIC, BIC, and DIC, are excellent ways to build models for prediction. However when such approaches are applied to a large number of potential predictors, the resulting models will produce some significant p values by chance alone. This arises even if model selection itself does not rely on p values. Thus it is critical to perform cross-validation before presenting model results, whether the goal is prediction (model fit) or inference (p values and confidence intervals). This can be done by splitting the data into a testing and training set, performing leave-one-out-cross-validation, or many other approaches [9]. However when the sample size is small, in general or relative to P, splitting the dataset will compromise already-limited power.

Additionally, note that model selection methods based on change-in-estimate approaches (whereby a variable is defined as a confounder if its inclusion in a multivariable model changes the main effect estimate by more than a threshold) are not appropriate for non-collapsible measures, including the odds ratio (OR) and hazard ratio. These measures will change in magnitude with adjustment for a variable even if that variable is not a confounder [12].

#### Overadjustment

Overadjustment can be thought of as the inverse of uncontrolled confounding. Uncontrolled confounding arises from failure to measure important confounders, or failure to fully adjust for them (due to errors in measurement, or model misspecification). Overadustment, conversely, is due to inappropriate inclusion of variables in a regression model. In general, this occurs when one of two variable types are adjusted for: colliders or mediators. Note that methodologists disagree on the definition of the term “overadjustment” [13].

**Fig 1:**
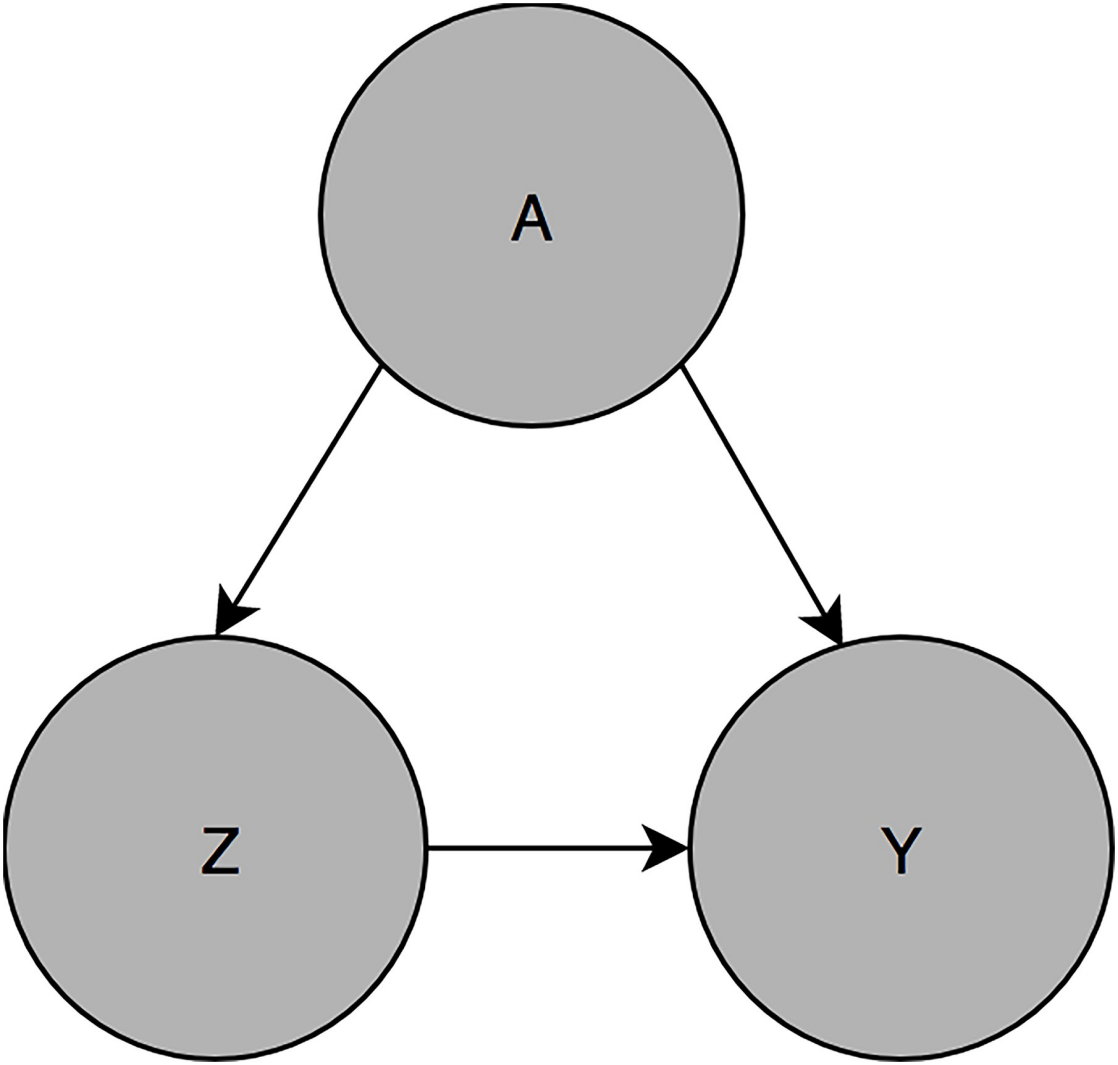
Structural representation of mediation and confounding Z and A both have causal effects of Y. A is a confounder of the Z-Y effect, and Z is a mediator of the A-Y effect.

While it is not uniformly inappropriate to model multiple exposures of interest in the same regression model, if one exposure is a confounder of the other, then that variable is in turn a mediator of the first. This is demonstrated in Fig 1: A is a confounder of the Z-Y relationship, and must be adjusted for to yield an unbiased estimate of the effect of Z on Y. However Z lies along the causal pathway from A to Y, and adjustment for Z will yield a *biased* estimate of the effect of A on Y. Thus Y should be regressed on both Z and A to estimate the Z effect, but to estimate the A effect Y should then be regressed on A in a separate model that does not include Z.

**Fig 2:**
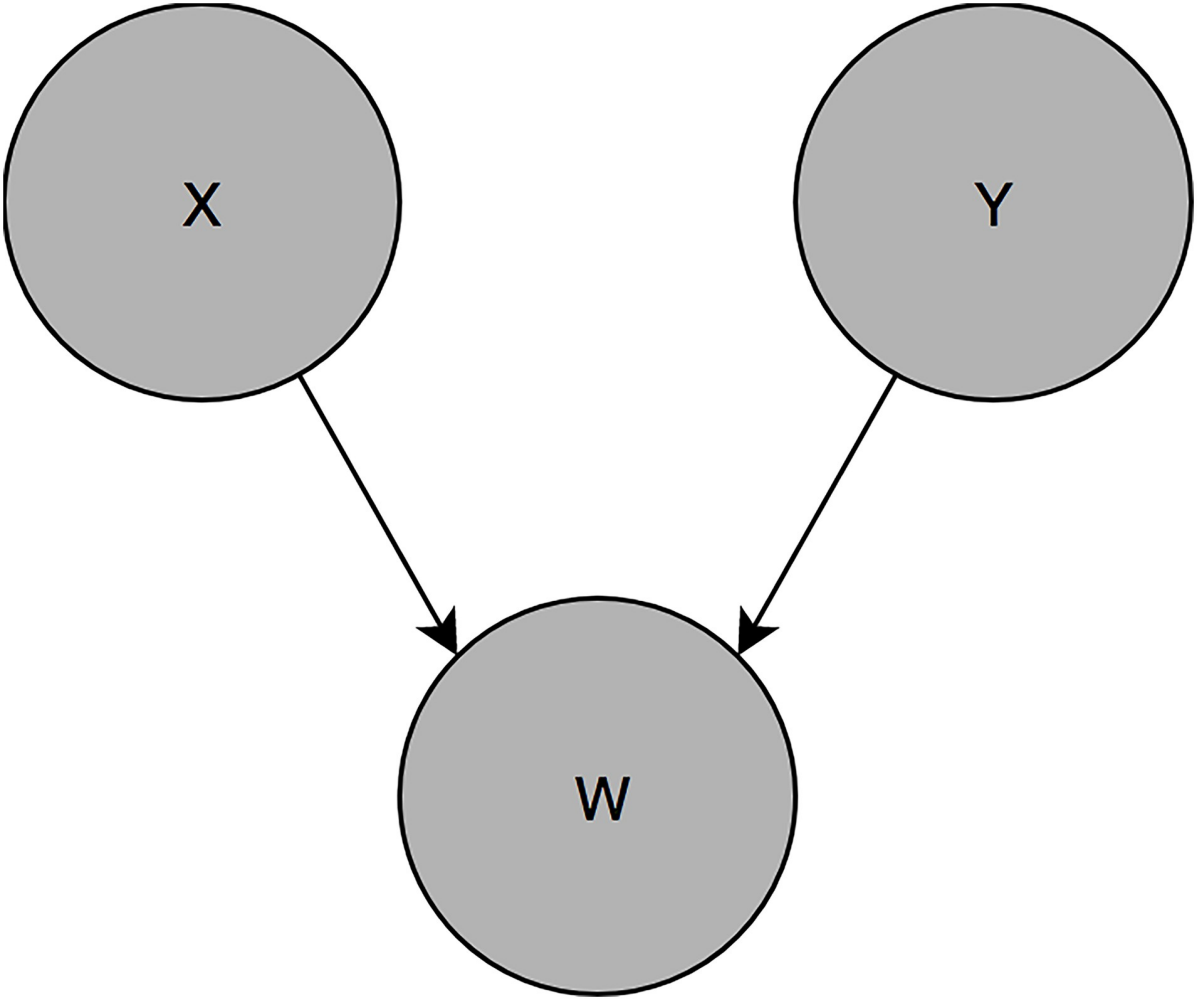
Structural representation of collider stratification bias X has no effect on Y. W is a descendant of X and Y, and a collider of the path from X to Y. Control for W will induce a spurious association between X and Y.

Collider stratification bias, also called Berkson’s bias, arises when the common effect of two variables is adjusted for. This will generate a spurious association between the two “parent” variables [12, 14]. The structure of this bias is presented in Fig 2: X has no effect on Y, demonstrated by the absence of an arrow from X to Y. However both cause W; control for W, either via a regression model or sampling conditional on W, will induce an association between X and Y. Note that if the goal of a study is purely prediction, it is not inappropriate to adjust for colliders or mediators. However if the goal is inference, including hypothesis generation or testing, these biased estimates interfere with that goal.

Unfortunately, bias due to overadjustment persists even in infinitely large samples [13]. Thus overadjustment is a threat no matter the dimensionality of the dataset. However it is avoided by approaches that carefully consider the role of each variable in a model, approaches which are labor-intensive to implement when P is large.

#### Matching: A potential hit to power

In a case-control study, matching refers to recruiting controls such that their distribution of important confounders reflects that of the cases. This causes bias towards the null in crude analyses, however when analyses appropriately reflect the matching design this bias is resolved, and in general results have greater statistical efficiency than unmatched designs [15].

Logistic regression models account for a matched design by either adjusting for matching variables or by performing conditional logistic regression. In the former, ORs are effectively estimated in groups that have equivalent values of all adjustment variables (“confounder strata”), while in the latter ORs are estimated within each matched set. The purpose of matching in case-control studies is therefore to improve statistical power, not to control for confounding: matching ensures there will not be confounder strata that contain only cases or only controls.

However, if matching or adjustment variables are so strongly associated with exposure that within a given confounder stratum or matched set nearly all observations have the same exposure value, then statistical power will be compromised by matching, contrary to its intent. If these variables are not strong confounders (strong risk factors for the outcome, as informed by subject-matter knowledge), this constitutes overmatching. Unlike inappropriate adjustment, which can be readily remedied during statistical analyses, inappropriate matching cannot be later rectified. While overmatching is not unique to low N:P data, unless a matching variable perfectly predicts exposure, the effects of overmatching diminish with increasing N:P ratios.

In general, matching on a factor makes the case and control populations more similar in all downstream factors. In these data, overmatching may arise if animal husbandry practices are downstream of (influenced by) village/neighborhood of residence, or if animal contact is downstream of (influenced by) the child’s age or sex, and if any one of these variables is not a strong risk factor for moderate-to-severe diarrhea.

### Simulation study

We first performed a simulation study to evaluate whether the findings of the GEMS-ZED substudy are indeed sensitive to overfitting, and whether p values produced by forward stepwise selection based on AIC are misleading when validation is not performed.

Using the data contained in the supplementary materials in Conan *et al*. [7] we simulated data for all 34 predictor variables for a cohort of 50,000, based on the empirical distribution of each variable among the controls. We did not generate the matching variables as data on their distribution was not available in this dataset. This means our simulated cohort is representative of the cases in terms of age, sex, and location distribution, but—if the outcome is rare and sampling is random—is otherwise representative of the super-population that cases and controls were sampled from. For categorical variables, we used the sample function to generate factor variables, setting the frequency of each level to that in the observed data. For binary variables we assumed a binomial distribution with probability of a value = 1 set to that in the observed data, using rbinom. For continuous variables we assumed a negative binomial distribution with mean and variance equal to the sample mean and sample variance, respectively, using rnegbin. As Conan *et al*. took measures to reduce the collinearity in these variables—collapsing variables where this was scientifically justified, and excluding the variable with the higher p-value otherwise [7]—and given the computational challenges expected with simulating from a 34 x 34 correlation matrix, we treated these variables as independent in our simulated data.

We then simulated an outcome under the crude model in Table 2 of Conan *et al*.:

$$\begin{array}{c} logit(P(Y=1))=log(0.4)\times \text{wash}\;\text{hands}+log(5)\times \text{rodent}\;\text{excreta}\\ +log(1)\times \text{sheep}+log(0.3)\times \text{sheep}\;\text{outside}\;\text{pen}\\ +log(2.1)\times \text{sheep}\;\text{in}\;\text{pen}+log(2.6)\times \text{chickens}\;\text{watering} \end{array}$$ (1)

where “wash hands” = child washes hands after contact with animals, “rodent excreta” = fresh rodent excreta (feces/urine) observed outside the house daily/often vs. seldom/never, “sheep” = total number of sheep, “sheep outside pen” = adult sheep sleep outside a pen, “sheep in pen” = adult sheep sleep in pen, and “chickens watering” = child’s presence during watering the chickens. We call this outcome “case.”

Next, we performed forward stepwise selection with “case” as the outcome using the steps function in R, with the lower limit equal to the empty model and the upper limit equal to the full model with all 34 predictors. All categorical variables were fit as dummy variables and all other variables as simple linear predictors. We performed this model selection on 100 simulated datasets, each a random draw of 73 cases and 73 controls from the full simulated cohort data. We evaluated consistency in model selection across these 100 simulated studies by calculating the number of models in which each predictor was included.

We then simulated a second outcome, which we will call “null case,” under the null model. That is, this outcome is independent of the predictors, and the *β*_1_ = … = *β*_*p*_ = 0 for all 34 predictors. We generated this outcome using the rbinom function, setting *p* equal to the prevalence of the first outcome (“case”). We drew our “training data” as a random case-control sample of 73 null cases and 73 controls and performed forward stepwise selection on this training data. Again, we fit categorical variables as dummy variables and all other variables as simple linear terms. We then drew a second random sample of 73 cases and 73, our “test data,” and fit the model selected on the training data to the test data.

### Proposed approach to regression modeling

To mitigate the risk of overadjustment bias associated with automated approach to variable selection, we propose taking an *a priori* approach. This is performed in four steps:

1. Identification of key exposures of interest
2. Identification of confounding variables for each exposure
3. Regression modeling for each exposure
4. Correction for multiple testing

Both steps (1) and (2) are motivated by existing subject matter knowledge. As dictated by the scientific method, a research question should be identified as before data are collected. Furthermore, as confounding is an inherently causal concept, study data can never be used to confirm or rule out confounding [16, 17]. Below we describe how we applied these steps to Conan *et al*.’s data, however these steps must be tailored to a given study’s research question. Thus the detailed methods we used, in particular the data cleaning steps, should not be interpreted as proscriptive.

#### 1. Identification of key exposures of interest

The GEMS-ZED dataset contains 797 predictor variables. To identify the key exposures of interest among these, we examined the full list of variables and grouped them as follows:

- Number and type of animals
- Presence of animals or their feces in the household cooking area
- Provision of nightshelter for animals
- Frequency of manure cleanup
- Manure use and storage
- Milk and egg consumption
- Water source and delivery for animal use
- Animal clinical signs
- Animal antibiotic use
- Deworming practices
- Child’s sleeping area proximity
- Habit of child playing in animals’ sleeping area or where animals defecate
- Direct contact between animal and child
- Child presence for husbandry tasks
- Child’s handwashing habits
- Preparation of milk and eggs for consumption

Within each group, we identified and retained variables of scientific interest (those deemed most relevant to the outcome of severe diarrhea), and performed several data cleaning steps. All variables pertaining to“other” species were dropped.

First, we collapsed all variables on animal sex and age (old vs. young), also retaining young animal-specific variables. Second, we re-coded ordinal categorical variables such that the lowest level (0) represented the lowest amount of animal contact. We re-coded all missing values to 0 if the total animal number for that species was non-missing and equaled 0.

Next, we collapsed across variables as follows. Animals entering, sleeping in, or defecating in the household cooking area collapsed to “cooking” (0: does not enter, 1: enters cooking area but doesn’t sleep or defecate, 2: sleeps but does not defecate, and 3: defecates). Animal shelter (pen or barn for livestock and donkeys/covered or uncovered in cage for poultry/kenneled or tied for dogs) collapsed to binary “housed.” History of anorexia, weight loss, reduced milk production (ruminants), staring haircoat (ruminants and donkeys) and ruffled feathers (poultry) collapsed to binary “ill.” Child playing where animal sleeps or defecates collapsed to binary “play,” Animal habit of nuzzling or licking child or child feeding or touching the animal collapsed to binary “nuzzle.contact.” Child’s presence for several husbandry variables collapsed: releasing or herding to “release.herd”, feeding or watering to “feed.water”, cleaning nightshelter or removing manure/feces to “cleaning”, and presence for slaughtering (livestock, donkeys, and poultry), butchering (livestock and donkeys) or skinning (poultry) to “dressing.” Finally, provision of housing and sheltering within the household living was collapsed to “nightshelter” (0: not housed, 1: housed not in living are, 2: housed in household living area).

We then collapsed within key categorical variables to generate binary variables: manure use (1: used on farm for livestock and donkeys/on farm for poultry/thrown in shamba for small animals, 0 otherwise), water delivery for animals (1: water brought or both animals brought and water delivered, 0: animals went to water), and eggs consumed untreated (1: neither boiled nor cooked, 0: boiled or cooked).

Finally, we summarized all variables over animal species: ruminants (cattle, sheep, goats), livestock (ruminants plus pigs), poultry (ducks, chickens, and turkeys where data were available), and small animals (dogs and cats). This was accomplished for continuous variables by summing across species, and for ordinal and binary variables by taking the maximum value across species. The cleaned dataset contained 624 variables.

We examined all summarized variables and identified exposures of interest for regression models based on our scientific interests. These exposures were:

- Total number of animals (young and adult; all, livestock, poultry, small animal; numeric)
- Animals defecate in cooking area (all, livestock, poultry, small animal; binary)
- Nightshelter (all, livestock, poultry, small animal; not housed/housed but not in household living area/housed in household living area)
- Frequency of manure cleanup (all, livestock, poultry, small animal; never/less than yearly/yearly/monthly/weekly/daily for livestock and poultry; never/seldom/often/daily for small animals)
- Manure used in house or farm (all, livestock, poultry; binary)
- Feces buried (small animals; binary)
- Milk or eggs from compound animals consumed within the past 3 weeks (binary)
- Milk or eggs consumed untreated (not boiled and not boiled or cooked, respectively; binary)
- Animal water source is the same as household’s (all, livestock, poultry, small animal; binary);
- Number with diarrhea in the past 3 weeks (all, livestock, poultry, small animal; numeric)
- Antibiotic use (all, livestock, poultry; never/as needed/routinely)
- Deworming frequency (small animal; never/less than yearly/yearly/6 monthly/3 monthly/monthly)
- Distance between child and animal sleeping areas (all, livestock, poultry, small animal; numeric)
- Child plays where animal sleeps or defecates (all, livestock, poultry, small animal; neither/sleeps or defecates/both)
- Child present for cleaning of nightshelter or removal of manure (all, livestock, poultry, small animal; binary)
- Child present for birthing (livestock; binary)
- Child present for dressing (all, livestock, poultry; slaughter, skinning, plucking, or butchering; binary)
- Child has direct contact with animal (all, livestock, poultry, small animal; neither/child feeds, pets, touches or animal nuzzles, licks/both)
- Child washes their hands after animal contact (binary).

#### 2. Identification of confounding variables for each exposure

Potential confounders for each exposure-outcome association were identified via construction of a Directed Acyclic Graph (DAG) in DAGitty.net S1 Fig. DAGs are a method of presenting hypothesized causal relationships [16], and can be used to identify the minimum sufficient adjustment set for each exposure-outcome pair. This is the smallest number of variables that generates an unbiased estimate of the exposure effect, if the DAG is true. DAGs are constructed using only subject matter knowledge. Note both Figs 1 and 2 are DAGs.

Two latent (and thus unmeasured) variables, socioeconomic status (SES) and herd health, were identified as confounders for several exposure-outcome associations. Total herd size and nightshelter provision, collapsed across all species, were assumed to be the most appropriate proxies of SES. Antibiotic use, deworming frequency, and number of animals with diarrhea, collapsed across all species, were taken as proxies of herd health. In addition to the exposures of interest and these latent variables, rodent and rodent excreta contact were evaluated as potential confounders. All confounders identified in a given minimum sufficient adjustment set were adjusted for in that exposure’s model, without any further selection. We have omitted HIV status from our DAG as the GEMS-ZED data do not contain this variable, and furthermore we believe HIV will only be associated with the exposures of interest via its association with SES, thus HIV status is more likely to be an effect modifier than a confounder.

#### 3. Regression modeling for each exposure

All variables that did not vary over the entire dataset (SD = 0) were discarded. All categorical variables were adjusted as grouped linear terms in all regression models. Conditional logistic regression models were fit for all exposures of interest, using the clogit function in the survival package in R [18]. No effect modification was evaluated.

Descriptive statistics were calculated for all exposures of interest. To evaluate the sensitivity of these data to overmatching, percent of discordant pairs for all binary variables was also calculated. With conditional logistic regression, only discordant case-control pairs (pairs in which the predictor is different for the case child and the control child) will contribute to the estimation of a given odds ratio.

We also imputed missing values in the exposures of interest using the multiple imputation by chained equation (MICE) approach in the mice package in R [19]. We imputed five datasets with 100 iterations, using classification and regression trees. All non-missing variables were used as predictors in the imputation model for a given variable with missingness. We repeated all conditional logistic regression models on the pooled imputed datasets, however we also present results on the complete case analyses (models fit to the dataset with missingness). Complete case analyses produce unbiased effect estimates if the pattern of missingness is completely at random (MCAR), that is if subjects with missing data would provide no additional information if their data were non-missing. Multiple imputation, on the other hand, produces unbiased effect estimates if the information contained in the missing data can be retrieved using information in the observed data, known as missing at random (MAR). While MCAR is a stronger assumption than MAR, we believe the imputation models are misspecified as there are few readily-available methods to impute missing data for matched case-control studies. We inspected trace plots to evaluate whether convergence was reached for each imputed variable, and examined density plots of imputed and observed data to ensure unreasonable values were not were imputed.

#### 4. Correction for multiple testing

As the 58 complete-case analyses correspond to 58 tested hypotheses, correction for multiple testing is warranted. We used the Benjamini-Hochberg approach, which is less conservative than a Bonferroni correction [20]. Only hypothesis tests on the exposures of interest were considered for this adjustment: if an exposure appeared in a second model as a potential confounder, this was not considered to be an additional hypothesis test. We also present uncorrected confidence intervals as we believe these are of greater use than p values.

### Alternative proposed approach: Latent variable modeling

In complement to our proposed regression approach, we also propose a latent variable modeling approach to the GEMS-ZED data. Latent variable modeling is a set of methods for modeling variables that cannot be directly measured. Latent variable modeling methods most familiar to epidemiologists include factor analysis and structural equations models, which are commonly used in the social sciences, in particular psychology. We propose instead the use of a method borrowed from test theory: Item Response Theory (IRT) [21]. IRT methods were developed to evaluate standardized tests such as the SATs or LSATs, and are suitable when the underlying latent trait is continuous and the items used to measure it are binary or categorical, as is the case for almost all of the GEMS-ZED predictors. We propose the true underlying latent trait to be animal contact. That is, we believe the items in the GEMS-ZED questionnaire measure animal contact.

We fit our IRT model to the cleaned dataset, after removing all variables specific to young animals (retaining variables collapsed over young and adult animals), all continuous variables (number of animals, distance between child’s and animals’ sleeping area, number of animals with diarrhea), all variables collapsed across species groups (all species, ruminants, livestock, poultry, and small animals) during the cleaning process, and all variables with standard deviation of 0. We also collapsed categorical variables into binary variables. We discarded the variables pertaining to the species of animal that the child’s milk and eggs came from and whether small animals are ever vs. never dewormed, as it is not clear which levels represent higher versus lower degrees of animal contact. Finally, we discarded variables pertaining to presence versus absence of animals of a given species, as these variables are more likely to be represent wealth than animal contact.

We fit the two-parameter logistic regression model (2PL) assuming a single latent trait, using the ltm package in R [22]. We modeled this latent trait as a simple linear term and presented a density plot of the modeled latent trait. We inspected the Item Characteristic Curves (ICC; plots of the latent variable’s value versus the probability of a 1 vs. 0 answer for a given item) for each item and examined the response patterns and estimated latent trait value for a random subset of participants. We evaluated the amount of information provided by these items over the range of the latent trait by examining the test information function visually and calculating the percent of information contained within a range latent trait values. Note that this method estimates the latent trait as a standardized variable, thus a value of 0 represents a child with “average” animal contact, and a value of 1 represents a child whose animal contact level is 1 standard deviation above the mean.

As the IRT model relies on the MAR assumption, we fit this model without multiple imputation. However, methods for evaluating the assumptions underlying the IRT model perform better without missingness, thus for evaluating the IRT model we imputed missing values for the items comprising the model, using the same methods described above. We applied this multiply-imputed dataset to estimate internal consistency, or how closely related the items are; unidimensionality, or the assumption that the items represent a single latent trait; and local independence, or the assumption that conditional on the latent trait items are no longer correlated. We evaluated internal consistency using Cronbach’s *α*, unidimensionality using a scree plot produced by the nFactors package [23], and local independence by extracting residuals from the 2PL model and examining a correlation matrix for the residuals from all items.

Finally, we extracted the estimated latent trait value for each subject and performed conditional logistic regression on a quadratic polynomial of this latent trait (a linear term and a squared term), to allow the animal contact-diarrhea relationship to be non-linear. We adjusted for total number of animals to approximate SES.

## Results

Of the 624 variables in the cleaned dataset, 530 had a standard deviation greater than 0. Most variables with *SD* = 0 were pig-related variables, which were mostly missing.

### Simulation study

Data were simulated for the 34 predictors and a cohort of 50,000 as described above. In forward stepwise selection applied to 100 random case-control samples (N = 146, P = 34), in which the crude model presented in Table 2 of Conan *et al*. [7] is true, the median number of models (out of 100) that a given variable was included in was 15, with an interquartile range of [0, 21]. Thus, the selected model varied markedly across each simulated case-control study.

Results of a model selected by forward stepwise selection performed on training data and evaluated in test data, under which the null model is true, are presented in Table 1. Test and training data each consisted of a random sample of 73 cases and 73 controls. Results from the training data suggest a significant effect for adult cats being present but not sleeping in the household living area, and a close to significant effect for cattle defecating in the cooking area within the past 3 weeks. When this model was fit to the test data, no effects were significant, consistent with the true model.

**Table 1:** Forward stepwise selection applied to test and training data.

	Training data		Test data	
	OR	p value	OR	p value
Bovine defecated in cooking area*	0.47	0.06	0.67	0.31
Child present during chicken butchering	1.80	0.12	0.61	0.17
Adult cats present but do not sleep in living area	2.24	0.04	1.37	0.41
Adult cats sleep in living area	1.74	0.23	1.53	0.36

*Within the past 3 weeks

### Ethics statement

This study used only pre-existing de-identified data, acquired via request to the GEMS Executive Committee of the University of Maryland School of Medicine.

### Regression analysis

Descriptive statistics for “total animal” variables are presented in Table 2, and full descriptive statistics (total animals, livestock, poultry, small animal) are presented in S1 Table. Case children were more likely to reside in households where animals were housed in the living area (98.6% vs 89% for all animals; 69.9% vs. 58.9% for small animals); where manure was cleaned weekly (21.0% vs. 9.59%) rather than monthly (28.8% vs. 43.8%); where poultry manure was used in the farm (79.5% vs. 67.1%); and where antibiotics were used routinely for livestock (53.4% vs. 46.5%). Case children were less likely to reside in households that shared a water source with its animals (56.2% vs. 78.1%;). Case children were also less likely to be present for dressing poultry (34.2% vs. 43.8%) and more likely to wash their hands after animal contact (63% vs. 46.6%). There were no other appreciable differences in cases and controls. Number of discordant pairs for binary variables was particularly low for any animals defecating in cooking area (9 discordant pairs), small animal feces buried (0), eggs consumed untreated (2), milk consumed untreated (0), any animal’s water source being the same as the household’s (8), and child’s presence for livestock birthing (6) and livestock dressing (4). Thus very few case-control pairs contributed to analyses including these variables (Table 3).

**Table 2:** Descriptive statistics for summarized variables.

Variable	Cases N = 73 n (%)	Controls N = 73 n (%)
Total animals*	26.5 (21.1)	25.4 (19.4)
Total young animals*	9.33 (9.31)	9.16 (9.14)
*Defecate in cooking area*	69 (94.5%)	64 (87.7%)
*Nightshelter*		
Housed	1 (1.37%)	2 (2.74%)
Not housed	0 (0.00%)	6 (8.22%)
Living area	72 (98.6%)	65 (89.0%)
*Frequency of manure cleanup*		
Less than yearly	1 (1.37%)	0 (0.00%)
Yearly	35 (47.9%)	34 (46.6%)
Monthly	21 (28.8%)	32 (43.8%)
Weekly	16 (21.9%)	7 (9.59%)
Daily	0 (0%)	0 (0%)
*Manure used*	69 (94.5%)	65 (89.0%)
*Milk consumed*	25 (34.2%)	21 (28.8%)
Missing	0 (0.00%)	1 (1.37%)
*Eggs consumed*	28 (38.4%)	30 (41.1%)
Missing	4 (5.48%)	5 (6.85%)
*Milk untreated*	0 (0.00%)	1 (1.37%)
Missing	47 (64.4%)	51 (69.9%)
*Eggs untreated*	27 (37.0%)	26 (35.6%)
Missing	45 (61.6%)	40 (54.8%)
*Water source same as household*	41 (56.2%)	57 (78.1%)
Missing	24 (32.9%)	10 (13.7%)
Number of animals with diarrhea*	1.75 (7.40)	1.10 (4.63)
*Antibiotic use*		
Never	8 (11.0%)	7 (9.59%)
As needed	0 (0.00%)	1 (1.37%)
Routinely	65 (89.0%)	65 (89.0%)
Distance between sleeping areas*	6.45 (13.9)	6.40 (13.7)
Missing	2 (2.74%)	0 (0%)
*Plays where animal sleeps or defecates*		
Neither	11 (15.1%)	11 (15.1%)
Sleeps or defecates	3 (4.11%)	4 (5.48%)
Both	59 (80.8%)	58 (79.5%)
*Present for cleaning of night shelter*	55 (75.3%)	51 (69.9%)
Present for birthing**	4 (5.48%)	4 (5.48%)
*Present for dressing*^†^	26 (35.6%)	32 (43.8%)
*Animal contact*		
Child feeds, pets, touches	21 (28.8%)	20 (27.4%)
Animal nuzzles, licks	14 (19.2%)	14 (19.2%)
Both	38 (52.1%)	39 (53.4%)
*Washes hands*	46 (63.0%)	34 (46.6%)
Missing	5 (6.85%)	7 (9.59%)

*Mean, SD;

**Defined for livestock only;

^†^Slaughter, skinning/plucking, butchering.

**Table 3:** Case-control pairs with non-missing and discordant exposures for binary variables.

Variable	# Pairs	% Pairs
*Animals defecate in cooking area*	9	12.33
Livestock	25	34.25
Poultry	14	19.18
Small animal	10	13.70
*Manure used*	12	16.44
Livestock	28	38.36
Poultry	27	36.99
Small animal*	0	0.00
*Eggs consumed*	27	36.99
*Milk consumed*	32	43.84
*Eggs untreated*	2	2.74
*Milk untreated*	0	0.00
*Water source same as household*	8	10.96
Livestock	34	46.58
Poultry	15	20.55
Small animal	22	30.14
*Present for feeding or cleaning*	20	27.40
Livestock	25	34.25
Poultry	27	36.99
Present for birthing**	6	8.22
*Present for dressing*^†^	32	43.84
Livestock	4	5.48
Poultry	33	45.21
*Plays where animal sleeps or defecates*	17	23.29
Livestock	27	36.99
Poultry	23	31.51
Small animal	28	38.36
*Animal contact*	37	50.68
Livestock	35	47.95
Poultry	40	54.79
Small animal	41	56.16
*Washes hands*	24	32.88

*Feces buried;

**Defined only for livestock;

^†^Slaughter, skinning/plucking, butchering.

Conditional logistic regression results for “total animal” variables are presented in Table 4, and full results are presented in S2 Table. The constructed DAG is presented in S1 Fig, and the footnotes of these tables indicate the minimum sufficient adjustment set identified from this DAG for each exposure of interest. Diagnostics—trace plots to evaluate convergence, and density plots for imputed and observed variables—for the multiple imputation are presented in S1 and S2 Files. Convergence appears to have been reached, without unreasonable imputed values. Results are presented for both the complete case analysis (MCAR assumption) and for regression on the pooled imputed datasets (MAR assumption).

**Table 4:** Regression results.

Variable	Complete case	Imputed dataset
	OR, 95% CI	
*Total* *animals*^a^	1 (0.98, 1.02)	1 (0.98, 1.02)
*Total* *young*^a^ *animals*	1 (0.96,1.04)	1 (0.96,1.04)
*Defecate in cooking area*^b^	0.45 (0.07,2.81)	0.45 (0.07,2.81)
*Nightshelter*^c^	0.3 (0.07,1.24)	0.3 (0.07,1.24)
*Frqeuency of manure cleanup*^d^	0.94 (0.57,1.56)	0.94 (0.57,1.56)
*Manure used*^e^	0.99 (0.23,4.32)	0.99 (0.23,4.32)
*Eggs consumed*^f^	1.24 (0.57,2.67)	1.17 (0.54,2.55)
*Milk consumed*^f^	0.87 (0.4,1.86)	0.93 (0.44,1.97)
*Water source same as household*^e^	2.75 (0.49,15.26)	2.82 (0.74,10.73)
*Number with diarrhea*^g^	0.98 (0.93,1.04)	0.98 (0.93,1.04)
*Antibiotic use*^h^	1.04 (0.58,1.86)	1.04 (0.58,1.86)
*Distance between sleeping areas*^i^	1 (0.97,1.03)	1 (0.98,1.03)
*Plays where animal sleeps or defecates*^j^	1.19 (0.60,2.35)	1.25 (0.63,2.47)
*Present for cleaning of nightshelter*^k^	0.55 (0.15,2.06)	0.7 (0.25,1.9)
Present for birthing*^,^^k^	1.04 (0.06,16.99)	1.08 (0.2,5.66)
*Present for dressing*^†^^,^^k^	3.2 (0.94,10.88)	1.55 (0.72,3.3)
*Animal contact*^l^	1.1 (0.65,1.85)	1.16 (0.7,1.93)
*Washes hands*^e^	0.49 (0.21,1.16)	0.43 (0.19,0.98)

*Defined only for livestock.

^†^Slaughter, skinning/plucking, butchering

^*a*^Minimum sufficient adjustment set (“set”): socioeconomic status (SES), however provision of nightshelter is a mediator and was not adjusted for; no adjustment performed.

^*b*^Set: SES, total number of animals, nightshelter; adjusted for total number of animals and nightshelter as proxies.

^*c*^Set: SES, total number of animals; adjusted for total number of animals.

^*d*^Set: Total number of animals, manure use, nightshelter; full set adjusted.

^*e*^Set: SES; adjusted for total number of animals and nightshelter as proxies.

^*f*^Set: SES, total number of animals; adjusted for total number of animals and nightshelter.

^*g*^Set: herd health; adjusted for antibiotic use and deworming as proxy.

^*h*^Set: herd health; adjusted for number (across all animals) with diarrhea as proxy.

^*i*^Set: SES, nightshelter; adjusted for total number of animals and nightshelter.

^*j*^Set: SES, total number of animals, child presence for husbandry tasks, frequency of manure cleanup, nightshelter, and sleeping area proximity; adjusted for total number of animals, child’s presence for feeding or cleaning, frequency of manure cleanup, nightshelter, and sleeping area proximity.

^*k*^Set: SES, total number of animals, nightshelter, water source; adjusted for total number of animals, nightshelter, and shared water source.

^*l*^Set: child’s presence for husbandry tasks, sleeping area proximity; full set adjusted.

None of the exposures evaluated were significantly associated with the outcome of moderate to severe diarrhea before or after Benjamini-Hochberg correction (Table 4). Livestock water source being the same as the household’s and child presence for poultry dressing were close to statistically significant (p values 0.06 for both), however after correction for multiple testing these p values were no longer close to significant. Interestingly, both of these exposures had markedly differently effect estimates under the MCAR vs. MAR assumptions. While effect estimates were moderately strong for several exposures (any animals or livestock defecating in cooking area, MCAR and MAR; nightshelter for any animals or small animals, MCAR or MAR; use of livestock, poultry or small animal manure, MCAR or MAR; frequency of poultry manure cleanup, MAR or MCAR; eggs or milk consumed, MCAR stronger than MAR; number of livestock with diarrhea, MCAR or MAR; antibiotic use in livestock or poultry, MCAR or MAR; dewormer use in small animals, MAR only; child presence for cleaning of the nightshelter of any animal, poultry, or small animal, MCAR or MAR; livestock contact, MAR or MCAR; and child washes hands, MAR or MCAR), confidence intervals were wide for all of these exposures. However, two variables had strong effect estimates and confidence intervals with relatively high lower bounds: playing where poultry sleep or defecate (MCAR and MAR; MAR estimate: OR 1.56, 95% CI 0.85, 2.84), and poultry water source same as household (MAR only; OR 2.76, 95% CI 0.73, 10.46).

### Latent variable analysis

**Fig 3:**
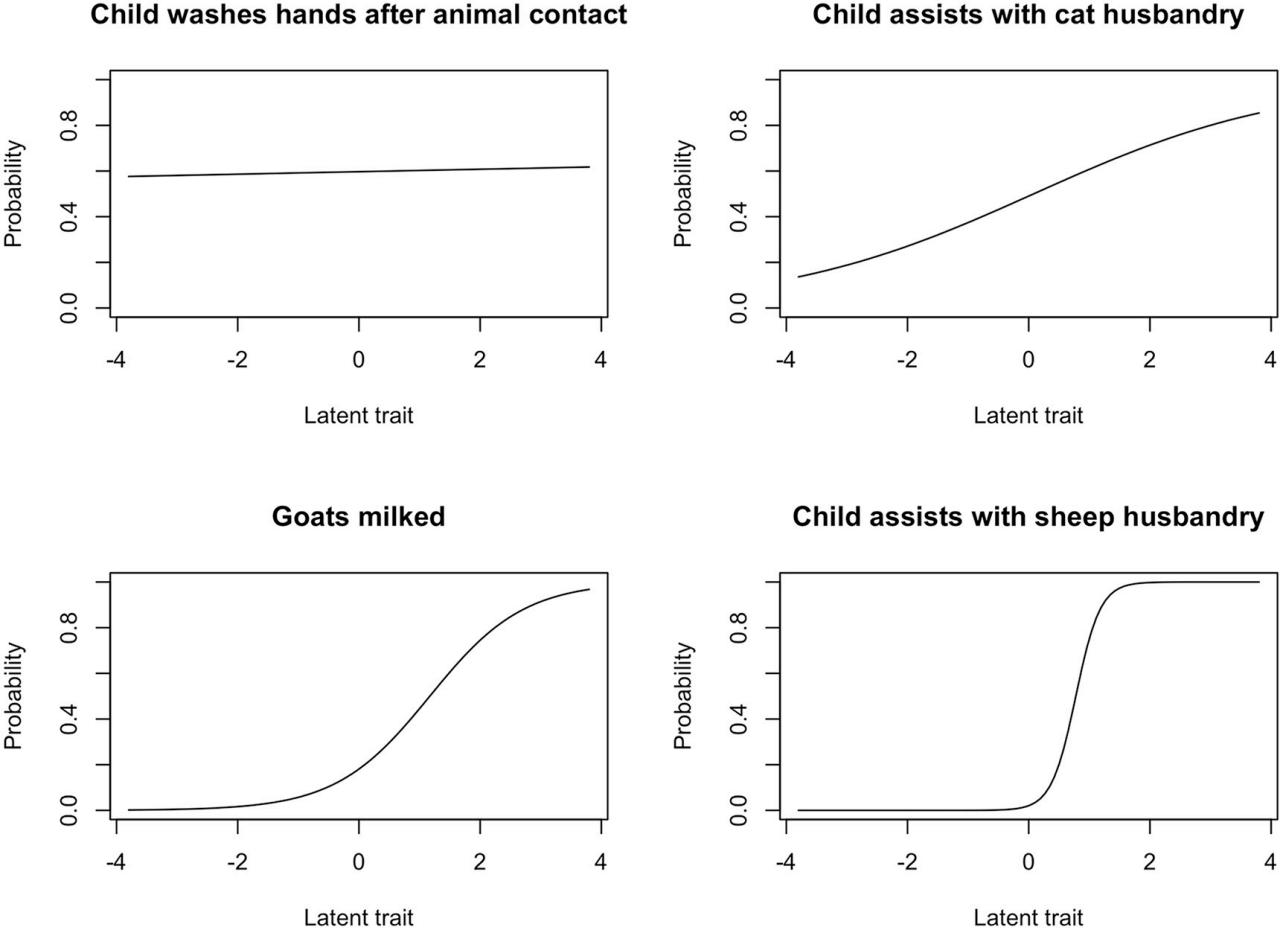
Item Characteristic Curves for four items. Child washes hands after animal contact: 1st quartile, discrimination = 0.02. Child assists with cat husbandry: 2nd quartile, discrimination = 0.48. Goats milked: 3rd quartile, discrimination = 1.29. Child assists with sheep husbandry: 4th quartile, discrimination 4.98. In test theory, discrimination values > 1 are considered good.

The variable pertaining to child’s presence for cattle dressing was removed as only one participant had value = 1 for this variable. The latent variable model was fit to the remaining 177 items and all 146 participants; Across these items, the median discrimination ability was 0.79, with an interquartile range of [0.12, 1.73]. Discrimination ability measures each item’s ability to distinguish between high versus low values of the latent trait, which we propose here to be animal contact. In test theory, discrimination values of greater than one are considered good. ICCs for four items, one from each quartile of discrimination ability, are presented in Fig 3. At the two lower quartiles of the distribution discrimination is poor, however discrimination is favorable at higher quartiles. ICC curves shifted to the right represent more “difficult” items, that is items for which the latent trait must take on a higher value for the respondent to answer “yes.”

**Fig 4:**
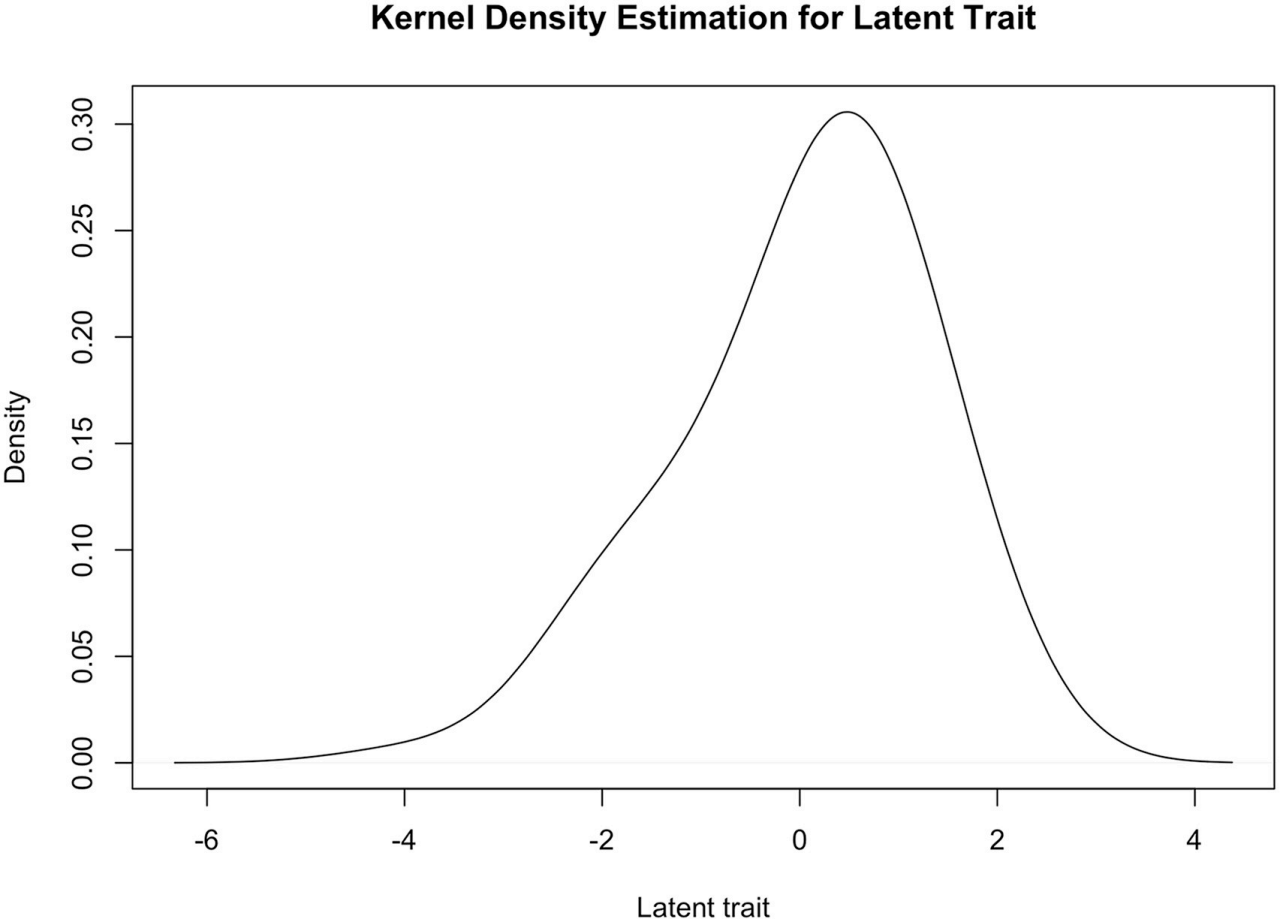
Density plot of latent trait values.

A density plot of the latent trait values is presented in Fig 4. Most participants have higher than mean values of the latent trait. Across the full range of the latent trait, 63.70% of the information contained in these items lay within [-2,2] standard deviations of the mean latent trait value; the full test information function is presented in S2 Fig. Cronbach’s *α*, calculated on the five pooled imputed datasets, was 0.942 (95% CI: 0.931, 0.951), indicating the 177 items had a high degree of internal consistency. A scree plot of the first 10 eigenvalues of the tetrachoric correlation matrix, computed on the first imputed dataset, is presented in S3 Fig, and suggests there is one dominant factor and possibly one smaller factor. To evaluate local independence, residuals for the 2PL model fit to the first imputed dataset were extracted and their correlations across all items inspected. Correlations were generally small (<0.5 in absolute value), however for the ruminant variables several strong correlations remained, namely: sheep milked was strongly correlated with child present for sheep husbandry (0.69), sheep manure ever removed (0.71), and sheep manure used in farm or house (0.73); goats milked was strongly correlated with goats sleep in cooking area (0.52); and cattle milked was strongly correlated with cattle milk consumed by household (0.98), any milk consumed by child (0.89), and cattle sleep in cooking area (0.77). Finally, response patterns from a random selection of 10 participants are presented in S3 Table.

On simple linear regression adjusting for total number of animals, the odds of moderate to severe diarrhea was 21% higher for a child with a 1 standard deviation higher value of the latent trait (95% CI 0.78, 1.87). On polynomial regression, the odds ratio for the linear term was 1.22 (95% CI 0.78, 1.89) and for the quadratic term was 1.04 (95% CI 0.85, 1.28). Neither of these effect estimates reached statistical significance.

## Discussion

Using simulation, we have demonstrated the risks of automated variable selection in datasets with a high P:N ratio, and the risks of presenting p values from a forward stepwise selected model without validation. Across the 100 simulated datasets, the variables selected for inclusion in each dataset varied markedly. Furthermore under the null model, forward stepwise selection performed on a training dataset produced significant findings that were not replicated in a test dataset. We propose two alternative approaches appropriate to this setting: an *a priori* model selection approach and latent variable modeling.

With our proposed *a priori* approach, none of the effect estimates reach statistical significance, however there was suggestive evidence of a trend for the livestock and poultry water sources being the same as the household’s, child presence for poultry dressing, and child playing where poultry sleep or defecate. These findings suggest that poultry exposure is a risk factor for moderate to severe diarrhea. This is perhaps unsurprising, as Conan *et al* [7] isolated non-typhoidal *Salmonella*—a known cause of pediatric diarrhea and a risk factor for death among hospitalized children [24]—from 11.3% of sampled chickens. Alternatively this trend could represent unmeasured confounding by wealth (if low wealth results in greater poultry contact), measurement error that is differential between cases and controls (possible if interviewers are not blinded to case-control status, or if interviewees from case households are more or less likely to answer questionnaire items accurately than those from control households), or selection bias. Selection bias, a form of collider stratification bias, is a particular risk in case-control studies as selection into the study is always downstream of the outcome of interest. Thus if selection is in any way downstream of the exposure of interest, collider stratification bias results from study design alone [25].

With regards to sharing of water with livestock and with poultry, it is difficult to comment on this finding using these data alone. If the animal is herded to the water source, contamination of the household’s water supply could result. However all respondents brought water to their poultry, and only 3 case-control pairs who herded their livestock to water were discordant on whether the household shared that water source. Again this effect could also represent confounding by wealth (as it is possible that households with lower wealth may be more likely to share a water source with their livestock), differential measurement error, or selection bias.

Our results were overall similar for the complete case analysis and analysis on the imputed datasets. This suggests the missingness pattern was generally either completely at random (MCAR) or not at random (MNAR). Under MNAR, the pattern of missingness depends on unobserved data, including potentially the value of the missing variable itself. By definition it is not possible to confirm or rule out MNAR missingness.

Again, it should be noted that the steps we followed for the *a priori* approach must be tailored to the research question (and practical constraints). Thus, the exact application of these steps will be unique to a given analysis. The key to the *a priori* approach is that it is motivated solely by background knowledge, and not by information contained in the data.

Conan *et al*.’s final multivariate model included hand washing, adult sheep sleeping in the pen (no adult sheep (ref) vs. adult sheep sleep outside pen vs. adult sheep sleep in pen), total number of sheep, fresh rodent excreta observed outside home (never/seldom vs. daily/often) and child’s presence during watering chicken. Hand washing was protective and observing fresh rodent excreta daily or often was a risk factor, as was child’s presence during watering the chickens. The results related to sheep contact are harder to interpret; first, it is possible that the adult sheep sleeping variable is a mediator of the total sheep—diarrhea pathway. Second, in analyses of animal contact, if categorical variables are recoded such that the reference level represents no animals of that species owned, it is not possible to then adjust for number of animals of that species owned. This may explain why “adult sheep sleep in pen” has a harmful effect prior to adjustment (OR = 2.1), and a protective effect following adjustment (OR = 0.6) (Table 2 [7]). Thus while our findings did not reach statistical significance, we feel the interpretability gained by the *a priori* approach remains a considerable advantage. As discussed above, p values and confidence intervals cannot be interpreted directly from models fit to the training data.

The results of the latent variable analysis suggest that the latent trait is a risk factor for pediatric diarrhea, however this effect did not reach statistical significance. IRT analyses make three assumptions: (1) monotonicity, or the assumption that as the latent trait increases, the probability of a “yes” response to a binary question increases; (2) Unidimensionality, or the assumption that there is one dominant latent trait being measured; (3) Local independence, or the assumption that conditional on the latent trait the responses given to separate items are independent. To address monotonicity, we coded all 177 items such that value = 1 corresponded to a higher degree of animal contact. To assess unidimensionality, we plotted the eigenvalues of the first 10 components. This plot suggests there is one dominant factor and possibly one smaller factor, however scree plots of eigenvalues of the tetrachoric correlation matrix may suggest too many factors [26]. We assessed local independence by evaluating item correlations among model residuals, and suggest that in general local independence appears satisfied, with the exception of a small number of ruminant-specific variables. This may suggest misspecification of the 2PL model, or a second smaller dimension of ruminant husbandry or management type.

We are not aware of any prior attempts to evaluate the performance of IRT models fit to matched case-control data. Matching is essentially a form of biased sample selection, in which the controls are artificially similar to the cases. In IRT, parameters—difficulty and discrimination—are invariant across samples, up to a linear transformation. This means that a given item’s properties are not specific to the population in which they were evaluated and that the sample (at least theoretically) does not need to be randomly selected [26]. However if the entire sample performs similarly on one item, the parameters for that item will have a high standard error; thus we believe that matched (biased) sample selection should not unduly influence the IRT model’s parameters, but suggest future simulation studies to explore this question.

The percent of discordant pairs was less than 50% for 29 out of 32 binary variables evaluated in our regression models. Likely the result of matching for variables that are upstream of the exposures of interest, this compromises the power of our findings, whether the matching variables are or are not strong confounders (overmatching). This loss of power may explain the lack of statistically significant results for both our *a priori* model selection and latent variable modeling approaches.

While the *a priori* approach is somewhat labor-intensive, this approach allows the effect of exposures of interest to be interpreted as they were measured. As these exposures may be more readily intervened on than latent variables, this is often desirable. Note the *a priori* approach may still result in multivariable models with high P:N ratios, and thus this approach is not immune to the effects of overfitting. However as the structure of all fitted models are determined by the user, implications of this overfitting will be limited to effect estimates and confidence intervals, not to the variables included in a given model. The latent variable approach, conversely, is more amenable to automation. Furthermore, the effect of the latent trait may be of primary scientific interest for questions of mechanism or etiology. In this setting, latent variable modeling may be preferable to our *a priori* approach. We suggest that these approaches are often complimentary.

## Conclusion

Rich datasets, like that generated by the GEMS-ZED substudy, provide the opportunity to answer many research questions in a single analysis. When the data were collected on a vulnerable or hard-to-access population, or the research questions are particularly sensitive, this opportunity should not be overlooked. However, there are analytical challenges attendant to the analyses of these datasets, which will not present themselves as errors in statistical software. To ensure the scientific value—and thus the public health impacts—of such analyses are optimized, we propose an *a priori* approach to variable selection, in complement with latent variable modeling if it is reasonable to hypothesize the presence of a latent trait and its effects are of scientific interest. We also urge caution in selection of matching variables; in particular, matching variables must be strong risk factors for the outcome of interest (determined by subject-matter knowledge), and care should be taken before matching for variables that may be strongly associated with the exposure of interest. If such variables are truly strong confounders, sample size should be increased accordingly, if at all possible.

## Supporting information

S1 Table Full descriptive statistics for regression variables. (PDF)

S2 Table Full regression results. (PDF)

S3 Table Selected response patterns. Response patterns and latent trait values for a random sample of 10 respondents.
(PDF)

S1 File Trace lines of the MICE algorithm. Trace plots for the mean and standard deviation of each imputed variable against the iteration number.
(PDF)

S2 File Density plots of observed and imputed data. Density plots for each variable imputed using MICE, with observed data in blue, and imputed data (5 datasets in total) in red.
(PDF)

S1 Fig Directed Acyclic Graph. Constructed using DAGitty.net. This DAG is additionally published on DAGitty.net: Published DAG; grey nodes are unmeasured or latent variables.
(TIF)

S2 Fig Test information function. Test information function from latent variable analysis, which shows how the information contained in these items changes at different levels of the latent trait. The information function has two peaks, one large peak approximately 1 standard deviations above the mean, and a smaller peak at approximately 2.5 standard deviations above the mean. This suggests these items are good for determining average to high levels of the latent trait.
(TIFF)

S3 Fig Scree plot. Scree plot of the first 10 eigenvalues of the tetrachoric correlation matrix to assess the dimensionality of the data. Note this correlation matrix was fit to a single imputed dataset. There is a large drop between the first and second eigenvalues, but another large drop between the second and third eigenvalues. This could be interpreted as either one or two dominant factors.
(TIFF)
