# Supplementary material for: The curse of dimensionality: Animal-related risk factors for pediatric diarrhea in western Kenya, and methods for dealing with a large number of predictors
Source: PLoS One. 2019 Apr 26;14(4):e0215982. doi: 10.1371/journal.pone.0215982 (PMC6485705; doi:10.1371/journal.pone.0215982)
Supplement: S1 Table — (PDF) [file pone.0215982.s001.pdf]

S1 Table: Full descriptive statistics for regression variables

Variable	Cases	Controls
	N=73	N=73
	n (%)	n (%)
Total animals[*]	26.5 (21.1)	25.4 (19.4)
Livestock	10.2 (13.4)	10.0 (13.4)
Poultry	14.3 (13.5)	13.0 (11.2)
Small animal	1.84 (1.55)	1.96 (2.22)
Total young[*] *animals*	9.33 (9.31)	9.16 (9.14)
Livestock	1.89 (2.89)	1.75 (2.62)
Poultry	7.25 (8.60)	7.07 (8.48)
Small animal	0.18 (0.54)	0.23 (0.87)
Defecate in cooking area	69 (94.5%)	64 (87.7%)
Livestock	27 (37.0%)	22 (30.1%)
Poultry	65 (89.0%)	63 (86.3%)
Small animals	5 (6.85%)	5 (6.85%)
Nightshelter		
Not housed	1 (1.37%)	2 (2.74%)
Housed	0 (0.00%)	6 (8.22%)
Living area	72 (98.6%)	65 (89.0%)
Livestock		
Not housed	17 (23.3%)	15 (20.5%)
Housed	0 (0.00%)	1 (1.37%)
Living area	56 (76.7%)	57 (78.1%)
Poultry		
Not housed	5 (6.85%)	5 (6.85%)
Housed	33 (45.2%)	32 (43.8%)
Living area	35 (47.9%)	36 (49.3%)
Small Animal		
Not housed	18 (24.7%)	29 (39.7%)
Housed	1 (1.37%)	1 (1.37%)
Living area	51 (69.9%)	43 (58.9%)
Missing	3 (4.11%)	0 (0.00%)
Frequency of manure cleanup		
Never	0 (0%)	0 (0.00%)
Less than yearly	1 (1.37%)	0 (0.00%)
Yearly	35 (47.9%)	34 (46.6%)
Monthly	21 (28.8%)	32 (43.8%)
Weekly	16 (21.9%)	7 (9.59%)
Daily	0 (0%)	0 (0%)
Livestock		
Never	16 (21.9%)	13 (17.8%)
Less than yearly	7 (9.59%)	12 (16.4%)
Yearly	29 (39.7%)	27 (37.0%)
Monthly	21 (28.8%)	21 (28.8%)
Weekly	0 (0%)	0 (0%)
Daily	0 (0%)	0 (0%)
Poultry		
Never	4 (5.48%)	2 (2.74%)
Less than yearly	0 (0%)	0 (0%)
Yearly	66 (90.4%)	70 (95.9%)

Monthly	2 (2.74%)	1 (1.37%)
Weekly	1 (1.37%)	0 (0.00%)
Daily	0 (0%)	0 (0%)
Small animal		
Never	33 (45.2%)	36 (49.3%)
Seldom	15 (20.5%)	12 (16.4%)
Often	9 (12.3%)	18 (24.7%)
Daily	16 (21.9%)	7 (9.59%)
Manure used	69 (94.5%)	65 (89.0%)
Livestock	55 (75.3%)	55 (75.3%)
Poultry	58 (79.5%)	49 (67.1%)
Small animal*	25 (34.2%)	22 (30.1%)
Milk consumed	25 (34.2%)	21 (28.8%)
Missing	0 (0.00%)	1 (1.37%)
Eggs consumed	28 (38.4%)	30 (41.1%)
Missing	4 (5.48%)	5 (6.85%)
Milk untreated	0 (0.00%)	1 (1.37%)
Missing	47 (64.4%)	51 (69.9%)
Eggs untreated	27 (37.0%)	26 (35.6%)
Missing	45 (61.6%)	40 (54.8%)
Water source same as household	41 (56.2%)	57 (78.1%)
Missing	24 (32.9%)	10 (13.7%)
Livestock	40 (54.8%)	48 (65.8%)
Poultry	54 (74.0%)	62 (84.9%)
Missing	3 (4.11%)	2 (2.74%)
Small animal	26 (35.6%)	36 (49.3%)
Missing	22 (30.1%)	8 (11.0%)
*Number of animals with diarrhea**	1.75 (7.40)	1.10 (4.63)
Livestock	0.40 (0.98)	0.25 (0.78)
Poultry	1.36 (7.37)	0.85 (4.62)
Small animal†	0 (0)	0 (0)
Antibiotic use		
Never	8 (11.0%)	7 (9.59%)
As needed	0 (0.00%)	1 (1.37%)
Routinely	65 (89.0%)	65 (89.0%)
Livestock		
Never	34 (46.6%)	39 (53.4%)
As needed	0 (0%)	0 (0%)
Routinely	39 (53.4%)	34 (46.6%)
Poultry		
Never	14 (19.2%)	11 (15.1%)
As needed	1 (1.37%)	1 (1.37%)
Routinely	58 (79.5%)	61 (83.6%)
Small animal deworming		
Never	19 (26.0%)	25 (34.2%)
Every 3 months	3 (4.11%)	4 (5.48%)
Monthly	51 (69.9%)	44 (60.3%)
*Distance between sleeping areas**	6.45 (13.9)	6.40 (13.7)
Missing	2 (2.74%)	0 (0%)
Livestock	17.8 (19.0)	19.9 (19.2)
Missing	17 (23.29%)	13 (17.81%)
Poultry	14.0 (20.2)	12.1 (19.4)

Missing	7 (9.59%)	12 (16.44%)
Small animal	10.4 (17.9)	11.0 (20.0)
Missing	18 (24.66%)	26 (35.62%)
Plays where animal sleeps or defecates		
Neither	11 (15.1%)	11 (15.1%)
Sleeps or defecates	3 (4.11%)	4 (5.48%)
Both	59 (80.8%)	58 (79.5%)
Livestock		
Neither	30 (41.1%)	31 (42.5%)
Sleeps or defecates	2 (2.74%)	4 (5.48%)
Both	41 (56.2%)	38 (52.1%)
Poultry		
Neither	15 (20.5%)	12 (16.4%)
Sleeps or defecates	4 (5.48%)	4 (5.48%)
Both	54 (74.0%)	57 (78.1%)
Small animal		
Neither	38 (52.1%)	39 (53.4%)
Sleeps or defecates	9 (12.3%)	8 (11.0%)
Both	26 (35.6%)	26 (35.6%)
Animal contact		
Child feeds, pets, touches	21 (28.8%)	20 (27.4%)
Animal nuzzles, licks	14 (19.2%)	14 (19.2%)
Both	38 (52.1%)	39 (53.4%)
Livestock		
Child feeds, pets, touches	43 (58.9%)	40 (54.8%)
Animal nuzzles, licks	18 (24.7%)	20 (27.4%)
Both	12 (16.4%)	13 (17.8%)
Poultry		
Child feeds, pets, touches	28 (38.4%)	28 (38.4%)
Animal nuzzles, licks	17 (23.3%)	20 (27.4%)
Both	28 (38.4%)	25 (34.2%)
Small animal		
Child feeds, pets, touches	36 (49.3%)	34 (46.6%)
Animal nuzzles, licks	14 (19.2%)	15 (20.5%)
Both	23 (31.5%)	24 (32.9%)
Present for cleaning of night shelter	55 (75.3%)	51 (69.9%)
Livestock	30 (41.1%)	31 (42.5%)
Poultry	47 (64.4%)	48 (65.8%)
Small animal	8 (11.0%)	11 (15.1%)
*Present for birthing***	4 (5.48%)	4 (5.48%)
Present for dressing	26 (35.6%)	32 (43.8%)
Livestock	2 (2.74%)	2 (2.74%)
Poultry	25 (34.2%)	32 (43.8%)
Washes hands	46 (63.0%)	34 (46.6%)
Missing	5 (6.85%)	7 (9.59%)

*Mean, SD; **Defined for livestock only; †No variance within dataset, not retained for regression models.
Note all ordinal categorical variables were set to the lowest level if the household owned 0 animals of that type.
