# Supplementary material for: The curse of dimensionality: Animal-related risk factors for pediatric diarrhea in western Kenya, and methods for dealing with a large number of predictors
Source: PLoS One. 2019 Apr 26;14(4):e0215982. doi: 10.1371/journal.pone.0215982 (PMC6485705; doi:10.1371/journal.pone.0215982)
Supplement: S2 Table — (PDF) [file pone.0215982.s002.pdf]

S2 Table: Full regression results

Variable	Complete case	Imputed dataset
	OR, 95% CI	
Total animals[a]	1 (0.98, 1.02)	1 (0.98, 1.02)
Livestock[a]	1 (0.97, 1.02)	1 (0.97, 1.02)
Poultry[a]	0.99 (0.96, 1.02)	0.99 (0.96, 1.02)
Small animal[a]	1.06 (0.87, 1.29)	1.06 (0.87, 1.29)
Total young animals[a]	1 (0.96,1.04)	1 (0.96,1.04)
Livestock[a]	0.97 (0.85,1.12)	0.97 (0.85,1.12)
Poultry[a]	1 (0.95,1.04)	1 (0.95,1.04)
Small animal[a]	1.1 (0.71,1.72)	1.1 (0.71,1.72)
Defecate in cooking area[b]	0.45 (0.07,2.81)	0.45 (0.07,2.81)
Livestock[b]	0.72 (0.32,1.65)	0.72 (0.32,1.65)
Poultry[b]	1.05 (0.29,3.77)	1.05 (0.29,3.77)
Small animal[b]	1.02 (0.29,3.58)	1.02 (0.29,3.58)
Nightshelter[c]	0.3 (0.07,1.24)	0.3 (0.07,1.24)
Livestock[c]	1.19 (0.76,1.87)	1.17 (0.76,1.81)
Small animal[c]	0.72 (0.48,1.08)	0.53 (0.25,1.14)
Poultry[c]	1.07 (0.57,2)	1.02 (0.55,1.89)
Frqeuency of manure cleanup[d]	0.94 (0.57,1.56)	0.94 (0.57,1.56)
Livestock[d]	1.13 (0.8, 1.59)	1.13 (0.8,1.59)
Poultry[d]	1.24 (0.48, 3.19)	1.24 (0.48,3.19)
Small animal[d]	0.87 (0.6, 1.27)	0.87 (0.6,1.27)
Manure used[e]	0.99 (0.23,4.32)	0.99 (0.23,4.32)
Livestock[e]	1.55 (0.63,3.79)	1.55 (0.63,3.79)
Poultry[e]	0.55 (0.23,1.28)	0.55 (0.23,1.28)
Small animal[*,e]	0.82 (0.36,1.86)	0.82 (0.36,1.86)
Eggs consumed[f]	1.24 (0.57,2.67)	1.17 (0.54,2.55)
Milk consumed[f]	0.87 (0.4,1.86)	0.93 (0.44,1.97)
Water source same as household[e]	2.75 (0.49,15.26)	2.82 (0.74,10.73)
Livestock[e]	3.05 (0.95,9.79)	1.4 (0.64,3.08)
Poultry[e]	1.04 (0.18,6.19)	2.76 (0.73,10.46)
Small animal[e]	1.34 (0.42,4.26)	0.84 (0.37,1.91)
Number of animals with diarrhea[**,g]	0.98 (0.93,1.04)	0.98 (0.93,1.04)
Livestock[h]	0.83 (0.56,1.21)	0.83 (0.56,1.21)
Poultry[h]	0.99 (0.93,1.04)	0.99 (0.93,1.04)
Antibiotic use[i]	1.04 (0.58,1.86)	1.04 (0.58,1.86)
Livestock[i]	0.84 (0.59,1.21)	0.84 (0.59,1.21)
Poultry[i]	1.28 (0.73,2.24)	1.28 (0.73,2.24)
Small animal[†,i]	0.93 (0.81,1.07)	0.67 (0.34,1.33)
Distance between sleeping areas[j]	1 (0.97,1.03)	1 (0.98,1.03)
Livestock[k]	1.01 (0.93,1.09)	1 (0.98,1.02)
Poultry[k]	0.96 (0.92,1.01)	0.99 (0.97,1.01)
Small animal[k]	0.98 (0.93,1.03)	1.01 (0.98,1.03)
Plays where animal sleeps or defecates[l]	1.19 (0.60,2.35)	1.25 (0.63,2.47)
Livestock[l]	1.01 (0.61,1.66)	1.08 (0.66,1.76)
Poultry[l]	1.5 (0.83,2.73)	1.56 (0.85,2.84)
Small animal[l]	1.01 (0.57, 1.79)	1.03 (0.58,1.8)

Presnt for cleaning of nightshelter[m]	0.55 (0.15,2.06)	0.7 (0.25,1.9)
Livestock[n]	1.15 (0.46,2.87)	1.15 (0.46,2.87)
Poultry[n]	1.25 (0.51,3.02)	1.25 (0.51,3.02)
Small animal[n]	1.51 (0.52,4.39)	1.51 (0.52,4.39)
Present for birthing[‡,m]	1.04 (0.06,16.99)	1.08 (0.2,5.66)
Present for dressing[m]	3.2 (0.94,10.88)	1.55 (0.72,3.3)
Livestock[★,o]	1.13 (0.15,8.71)	1.13 (0.15,8.71)
Poultry[★,m]	3.2 (0.94,10.88)	1.62 (0.77,3.43)
Animal contact[p]	1.1 (0.65,1.85)	1.16 (0.7,1.93)
Livestock[p]	1.23 (0.72,2.11)	1.24 (0.73,2.11)
Poultry[p]	0.87 (0.52,1.45)	0.97 (0.59,1.58)
Small animal[p]	1.05 (0.67,1.63)	1.06 (0.69,1.63)
Washes hands[e]	0.49 (0.21,1.16)	0.43 (0.19,0.98)

[*]Feces buried. [**]Small animal variant had SD=0 and was dropped [†]Deworming. [‡]Defined only for livestock. Species group-specific models were fit livestock, poultry, and small animal variates in the same model unless otherwise indicated.

[★]Poultry and livestock fit in separate models due to convergence failure otherwise.

[a]Minimum sufficient adjustment set ("set"): socioeconomic status (SES), however provision of nightshelter is a mediator and was not adjusted for; no adjustment performed.

[b]Set: SES, total number of animals, nightshelter; adjusted for total number of animals and nightshelter as proxies.

[c]Set: SES, total number of animals; adjusted for total number of animals.

[d]Set: Total number of animals, manure use, nightshelter; full set adjusted.

[d]Set: SES; adjusted for total number of animals and nightshelter as proxy.

[f]Set: SES, total number of animals; adjusted for total number of animals and nightshelter.

[g]Set: herd health; adjusted for antibiotic use and deworming as proxy.

[h]Set: herd health; adjusted for antibiotic use as proxy.

[i]Set: herd health; adjusted for number (across all animals) with diarrhea as proxy.

[j]Set: SES, nightshelter; adjusted for total number of animals and nightshelter.

[k]Set: SES, nightshelter; adjusted for total number of animals, as model would not converge with adjustment for nightshelter.

[l]Set: SES, total number of animals, child presence for husbandry tasks, frequency of manure cleanup, nightshelter, and sleeping area proximity; adjusted for total number of animals, child's presence for feeding or cleaning, frequency of manure cleanup, nightshelter, and sleeping area proximity

[m]Set: SES, total number of animals, nightshelter, water source; adjusted for total number of animals, nightshelter, and shared water source.

[n]Set: SES, total number of animals, nightshelter, water source; adjusted for total number of animals and nightshelter, as model would not converge with adjustment for shared water source.

[o]Set: SES, total number of animals, nightshelter, water source; adjusted for total number of animals, as model would not converge with adjustment for nightshelter or shared water source.

[p]Set: child's presence for husbandry tasks, sleeping area proximity; full set adjusted.
