# Supplementary material for: The curse of dimensionality: Animal-related risk factors for pediatric diarrhea in western Kenya, and methods for dealing with a large number of predictors
Source: PLoS One. 2019 Apr 26;14(4):e0215982. doi: 10.1371/journal.pone.0215982 (PMC6485705; doi:10.1371/journal.pone.0215982)
Supplement: S3 Table — Response patterns and latent trait values for a random sample of 10 respondents. (PDF) [file pone.0215982.s003.pdf]

S3 Table: Selected response patterns

	Child 1	Child 2	Child 3	Child 4	Child 5	Child 6	Child 7	Child 8	Child 9	Child 10
Latent trait	1.60	2.54	1.79	0.23	-5.01	0.29	1.65	-1.68	0.29	2.10
Cows milked	0	0	0	0	0	0	1	1	1	1
Goats milked	0	0	0	0	0	1	0	1	0	1
Sheep milked	0	0	0	0	0	0	0	1	0	1
Chickens producing eggs*	0	1	1	1	1	1	1	1	0	1
Ducks producing eggs*	0	0	0	0	1	0	0	0	0	0
Cattle with diarrhea*	0	0	0	0	0	0	0	0	1	1
Goats with diarrhea*	0	0	0	0	0	0	0	0	0	1
Sheep with diarrhea*	0	0	0	0	0	0	0	0	0	0
Chickens with diarrhea*	0	0	0	0	0	1	0	0	0	0
Cattle antibiotics given more than “never”**	0	0	0	1	0	0	1	1	0	1
Goats antibiotics given more than “never”**	0	0	0	0	0	1	0	0	0	0
Sheep antibiotics given more than “never”**	0	0	0	0	0	0	0	0	0	0
Donkeys antibiotics given more than “never”**	0	0	0	0	0	0	0	0	0	0
Chicken antibiotics given more than “never”**	0	0	NA	0	0	0	0	0	0	0
Duck antibiotics given more than “never”**	0	0	0	0	0	0	0	0	0	0
Cattle manure removed more than “never”	0	0	0	1	0	0	1	1	1	1
Goats manure removed more than “never”	0	0	0	0	0	1	0	1	1	1
Sheep manure removed more than “never”	0	0	0	0	0	0	0	1	0	1
Donkey manure removed more than “never”	0	0	0	0	0	0	0	1	0	0
Chicken manure removed more than “never”	1	1	1	1	1	1	1	1	1	1
Duck manure removed more than “never”	0	0	0	0	1	0	0	0	0	0
Cattle sleep in cooking area	0	0	0	0	0	0	0	0	1	1
Goats sleep in cooking area	0	0	0	0	0	1	0	1	1	1
Sheep sleep in cooking area	0	0	0	0	0	0	0	0	0	0
Chickens sleep in cooking area	1	NA	1	1	1	1	1	NA	1	1
Ducks sleep in cooking area	0	0	0	0	1	0	0	0	0	0
Dogs sleep in cooking area	1	0	1	1	1	NA	0	1	0	1
Cattle enter cooking area	0	0	0	0	0	0	0	1	0	0
Goats enter cooking area	0	0	0	0	0	0	0	0	0	0
Sheep enter cooking area	0	0	0	0	0	0	0	0	0	0
Donkeys enter cooking area	0	0	0	0	0	0	0	0	0	0
Chickens enter cooking area	1	1	1	1	1	1	1	1	1	1
Ducks enter cooking area	0	0	0	0	0	0	0	0	0	0
Dogs enter cooking area	1	0	0	1	1	NA	0	1	0	1
Cats enter cooking area	NA	0	0	1	1	1	0	1	1	0

	Child 1	Child 2	Child 3	Child 4	Child 5	Child 6	Child 7	Child 8	Child 9	Child 10
Latent trait	1.60	2.54	1.79	0.23	-5.01	0.29	1.65	-1.68	0.29	2.10
Cattle defecate in cooking area	0	0	0	0	0	0	0	1	0	0
Goats defecate in cooking area	0	0	0	0	0	0	0	0	0	0
Sheep defecate in cooking area	0	0	0	0	0	0	0	0	0	0
Chickens defecate in cooking area	1	1	1	1	1	1	1	1	1	1
Ducks defecate in cooking area	0	0	0	0	0	0	0	0	0	0
Dogs defecate in cooking area	0	0	0	0	1	NA	0	1	0	0
Chickens defecate in cooking area	NA	0	0	0	0	0	0	0	0	0
Cattle sleep in living area	0	0	0	1	0	0	1	1	1	1
Goats sleep in living area	0	0	0	0	0	1	0	1	1	1
Chickens sleep in living area	0	1	0	1	0	0	0	1	0	0
Dogs sleep in living area	1	0	1	1	1	NA	0	1	0	1
Cats sleep in living area	NA	0	0	1	1	1	0	1	1	0
Cattle housed in pen or barn	0	0	0	1	0	0	1	1	1	1
Goats housed in pen or barn	0	0	0	0	0	1	0	1	1	1
Sheep housed in pen or barn	0	0	0	0	0	0	0	1	0	1
Donkeys housed in pen or barn	0	0	0	0	0	0	0	1	0	0
Chickens caged overnight, covered or uncovered	1	1	1	1	1	1	1	1	1	1
Ducks caged overnight, covered or uncovered	0	0	0	0	0	0	0	0	0	0
Dogs kenneled or tied in compound overnight	1	0	1	1	1	NA	0	1	0	1
Cattle manure used in farm or house	0	0	0	1	0	0	1	0	1	1
Goats manure used in farm or house	0	0	0	0	0	1	0	1	1	1
Sheep manure used in farm or house	0	0	0	0	0	0	0	1	0	1
Donkeys manure used in farm or house	0	0	0	0	0	0	0	0	0	0
Chicken manure used in the farm	0	1	1	1	1	1	1	NA	1	1
Duck manure used in the farm	0	0	0	0	0	0	0	0	0	0
Cattle manure stored	0	0	0	1	0	0	0	0	0	0
Goat manure stored	0	0	0	0	0	1	0	1	1	0
Sheep manure stored	0	0	0	0	0	0	0	1	0	0
Donkey manure stored	0	0	0	0	0	0	0	0	0	0
Chicken manure stored	0	0	0	0	0	0	0	0	0	0
Duck manure stored	0	0	0	0	NA	0	0	0	0	0
Dog feces buried	0	0	NA	0	0	NA	0	1	0	0
Cat feces buried	NA	0	0	0	1	0	0	0	0	0
Cow milk consumed	0	0	0	0	0	0	1	1	1	1
Goat milk consumed	0	0	0	0	0	0	0	0	0	0
Chicken eggs consumed	0	0	1	1	0	1	1	1	0	0
Duck eggs consumed	0	0	0	0	1	0	0	0	0	0

	Child 1	Child 2	Child 3	Child 4	Child 5	Child 6	Child 7	Child 8	Child 9	Child 10
Latent trait	1.60	2.54	1.79	0.23	-5.01	0.29	1.65	-1.68	0.29	2.10
Cattle water brought†	0	0	0	0	0	0	0	0	0	0
Goat water brought†	0	0	0	0	0	1	0	0	1	0
Sheep water brought†	0	0	0	0	0	0	0	0	0	0
Chicken water brought†	1	1	1	1	1	1	1	1	1	1
Duck water brought†	0	0	0	0	1	0	0	0	0	0
Dog water brought†	1	0	1	1	1	NA	0	1	0	1
Cat water brought†	NA	0	0	1	0	1	0	0	1	0
Cattle illness‡	0	0	0	0	0	0	1	1	1	1
Goat illness‡	0	0	0	0	0	0	0	1	1	0
Sheep illness‡	0	0	0	0	0	0	0	0	0	0
Chicken illness‡	0	0	0	1	0	1	0	1	0	0
Cattle/child sleeping area unseparated	1	1	1	0	1	1	0	0	0	0
Goat/child sleeping area unseparated	1	1	1	1	1	0	1	0	0	0
Sheep/child sleeping area unseparated	1	1	1	1	1	1	1	0	1	0
Donkey/child sleeping area unseparated	1	1	1	1	1	1	1	0	1	1
Chicken/child sleeping area unseparated	0	0	0	0	0	0	0	0	0	0
Duck/child sleeping area unseparated	1	1	1	1	0	1	1	1	1	1
Dog/child sleeping area unseparated	0	1	0	0	0	NA	1	0	1	0
Cat/child sleeping area unseparated	NA	1	1	1	1	1	1	1	1	1
Child plays where cattle sleep	0	0	0	1	0	0	1	1	0	1
Child plays where goats sleep	0	0	0	0	0	1	0	0	0	1
Child plays where sheep sleep	0	0	0	0	0	0	0	0	0	1
Child plays where donkeys sleep	0	0	0	0	0	0	0	1	0	0
Child plays where chickens sleep	1	1	0	1	1	1	1	1	0	1
Child plays where ducks sleep	0	0	0	0	0	0	0	0	0	0
Child plays where dogs sleep	1	0	0	1	1	NA	0	1	0	1
Child plays where cats sleep	NA	0	0	0	0	0	0	0	0	0
Child plays where cattle defecate	0	0	0	1	0	0	1	1	0	1
Child plays where goats defecate	0	0	0	0	0	1	0	0	1	1
Child plays where sheep defecate	0	0	0	0	0	0	0	0	0	1
Child plays where donkeys defecate	0	0	0	0	0	0	0	1	0	0
Child plays where chicken defecate	1	1	0	1	1	1	1	1	0	1
Child plays where ducks defecate	0	0	0	0	1	0	0	0	0	0
Child plays where dogs defecate	1	0	0	1	1	NA	0	1	0	1
Child plays where cats defecate	NA	0	0	0	0	0	0	0	0	0
Child feeds or touches cattle	0	0	0	0	0	0	0	1	0	0

	Child 1	Child 2	Child 3	Child 4	Child 5	Child 6	Child 7	Child 8	Child 9	Child 10
Latent trait	1.60	2.54	1.79	0.23	-5.01	0.29	1.65	-1.68	0.29	2.10
Child feeds or touches goats	0	0	0	0	0	1	0	1	0	0
Child feeds or touches sheep	0	0	0	0	0	0	0	1	0	0
Child feeds or touches donkeys	0	0	0	0	0	0	0	0	0	0
Child feeds or touches chickens	1	1	0	1	1	1	0	1	0	1
Child feeds or touches ducks	0	0	0	0	1	0	0	0	0	0
Child feeds or touches dogs	1	0	0	1	0	NA	0	1	0	1
Child feeds or touches cats	NA	0	0	1	1	1	0	1	1	0
Cattle nuzzle or lick child	0	0	0	0	0	0	0	0	0	0
Goats nuzzle or lick child	0	0	0	0	0	1	0	0	0	0
Sheep nuzzle or lick child	0	0	0	0	0	0	0	0	0	0
Chickens nuzzle or lick child	1	0	0	1	1	1	0	0	0	1
Ducks nuzzle or lick child	0	0	0	0	1	0	0	0	0	0
Dogs nuzzle or lick child	1	0	0	1	1	NA	0	0	0	1
Cats nuzzle or lick child	NA	0	0	1	0	0	0	0	1	0
Child assists in cattle husbandry	0	0	0	1	0	0	1	1	1	1
Child assists in goat husbandry	0	0	0	0	0	1	0	1	1	1
Child assists in sheep husbandry	0	0	0	0	0	0	0	1	0	1
Child assists in donkey husbandry	0	0	0	0	0	0	0	1	0	0
Child assists in chicken husbandry	1	1	1	1	1	1	1	1	1	1
Child assists in duck husbandry	0	0	0	0	1	0	0	0	0	0
Child assists in dog husbandry	1	0	NA	1	1	NA	0	1	0	1
Child assists in cat husbandry	NA	0	0	1	1	1	0	1	1	0
Child assists in releasing or herding cattle	0	0	0	1	0	0	0	1	0	0
Child assists in releasing or herding goats	0	0	0	0	0	1	0	1	0	0
Child assists in releasing or herding sheep	0	0	0	0	0	0	0	1	0	0
Child assists in releasing or herding donkeys	0	0	0	0	0	0	0	0	0	0
Child assists in releasing or herding chickens	0	0	0	1	1	1	1	0	1	0
Child assists in releasing or herding ducks	0	0	0	0	0	0	0	0	0	0
Child assists in feeding or watering cattle	0	0	0	1	0	0	0	0	0	0
Child assists in feeding or watering goats	0	0	0	0	0	1	0	0	0	0
Child assists in feeding or watering sheep	0	0	0	0	0	0	0	0	0	0
Child assists in feeding or watering donkeys	0	0	0	0	0	0	0	0	0	0
Child assists in feeding or watering chickens	1	1	1	1	1	1	1	0	1	0
Child assists in feeding or watering ducks	0	0	0	0	0	0	0	0	0	0
Child assists in feeding or watering dogs	0	0	1	1	1	NA	0	1	0	0
Child assists in feeding or watering cats	NA	0	0	0	1	1	0	1	1	0

	Child 1	Child 2	Child 3	Child 4	Child 5	Child 6	Child 7	Child 8	Child 9	Child 10
Latent trait	1.60	2.54	1.79	0.23	-5.01	0.29	1.65	-1.68	0.29	2.10
Child present for cleaning cattle nightshelter/manure removal	0	0	0	0	0	0	0	0	0	0
Child present for cleaning goat nightshelter/manure removal	0	0	0	0	0	1	0	0	0	0
Child present for cleaning sheep nightshelter/manure removal	0	0	0	0	0	0	0	0	0	0
Child present for cleaning donkeys nightshelter/manure removal	0	0	0	0	0	0	0	0	0	0
Child present for cleaning chicken nightshelter/manure removal	1	0	1	0	1	1	1	1	1	1
Child present for cleaning ducks nightshelter/manure removal	0	0	0	0	0	0	0	0	0	0
Child present for cleaning dog nightshelter/manure removal	0	0	0	1	0	NA	0	0	0	0
Child present for cleaning cat nightshelter/manure removal	NA	0	0	0	0	0	0	0	0	0
Child present during cattle milking	0	0	0	0	0	0	0	0	0	0
Child present during goat milking	0	0	0	0	0	0	0	0	0	0
Child present during collection of chicken eggs	1	0	0	1	0	0	1	0	1	1
Child present during collection of duck eggs	0	0	0	0	0	0	0	0	0	0
Child present when cattle are giving birth	0	0	0	0	0	0	0	0	0	0
Child present when goats are giving birth	0	0	0	0	0	0	0	0	0	0
Child present when sheep are giving birth	0	0	0	0	0	0	0	0	0	0
Child present when dogs are giving birth	0	0	0	0	0	NA	0	0	0	0
Child present when cats are giving birth	NA	0	0	0	0	0	0	0	0	0
Child present for vaccinating/deworming cattle	0	0	0	0	0	0	0	0	0	0
Child present for vaccinating/deworming goats	0	0	0	0	0	0	0	0	0	0
Child present for vaccinating/deworming sheep	0	0	0	0	0	0	0	0	0	0
Child present for vaccinating chickens	0	0	0	0	0	NA	0	0	0	0
Child present for vaccinating ducks	0	0	0	0	0	0	0	0	0	0
Child present for vaccinating/deworming dogs	0	0	0	0	0	NA	0	0	0	0
Child present for vaccinating/deworming cats	NA	0	0	0	0	0	0	0	0	0
Child present for goat dressing*	0	0	0	0	0	0	0	0	0	0
Child present for sheep dressing*	0	0	0	0	0	0	0	0	0	0
Child present for chicken dressing*	0	0	1	0	0	1	1	1	1	0
Child present for duck dressing*	0	0	0	0	0	0	0	0	0	0
Milk not boiled/cooked before child consumes	NA	NA	NA	NA	NA	NA	0	0	NA	0

	Child 1	Child 2	Child 3	Child 4	Child 5	Child 6	Child 7	Child 8	Child 9	Child 10
Latent trait	1.60	2.54	1.79	0.23	-5.01	0.29	1.65	-1.68	0.29	2.10
Eggs not boiled/cooked before child consumes	NA	1	NA	1	1	1	0	1	1	NA
Child consumed eggs*	0	1	NA	1	1	1	1	1	1	0
Child consumed milk*	0	0	0	0	0	0	1	1	0	1
Child washes hands after animal contact	0	0	1	0	1	1	1	0	1	0

"NA" = missing; *In the past three weeks; **Orally, in feed, or by injection. †Water brought to animals, or both animal goes to water and water brought to animals; ‡Anorexia, weight loss, reduced milk production (ruminants), staring haircoat (ruminants and donkeys), ruffled feathers (poultry); ⋆Slaughtering, butchering, or skinning
