# Supplementary figures and images for: The curse of dimensionality: Animal-related risk factors for pediatric diarrhea in western Kenya, and methods for dealing with a large number of predictors
Source: PLoS One. 2019 Apr 26;14(4):e0215982. doi: 10.1371/journal.pone.0215982 (PMC6485705; doi:10.1371/journal.pone.0215982)

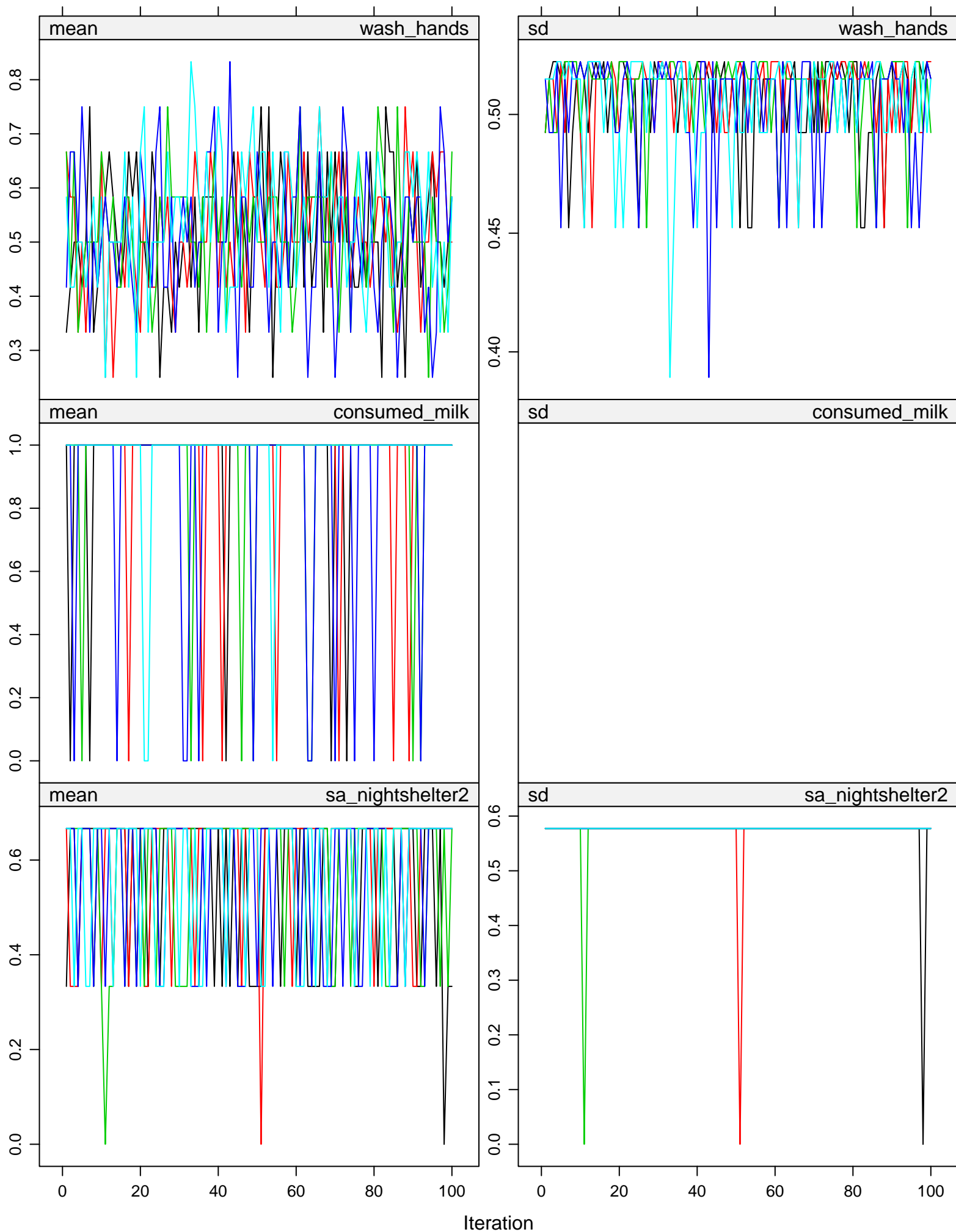

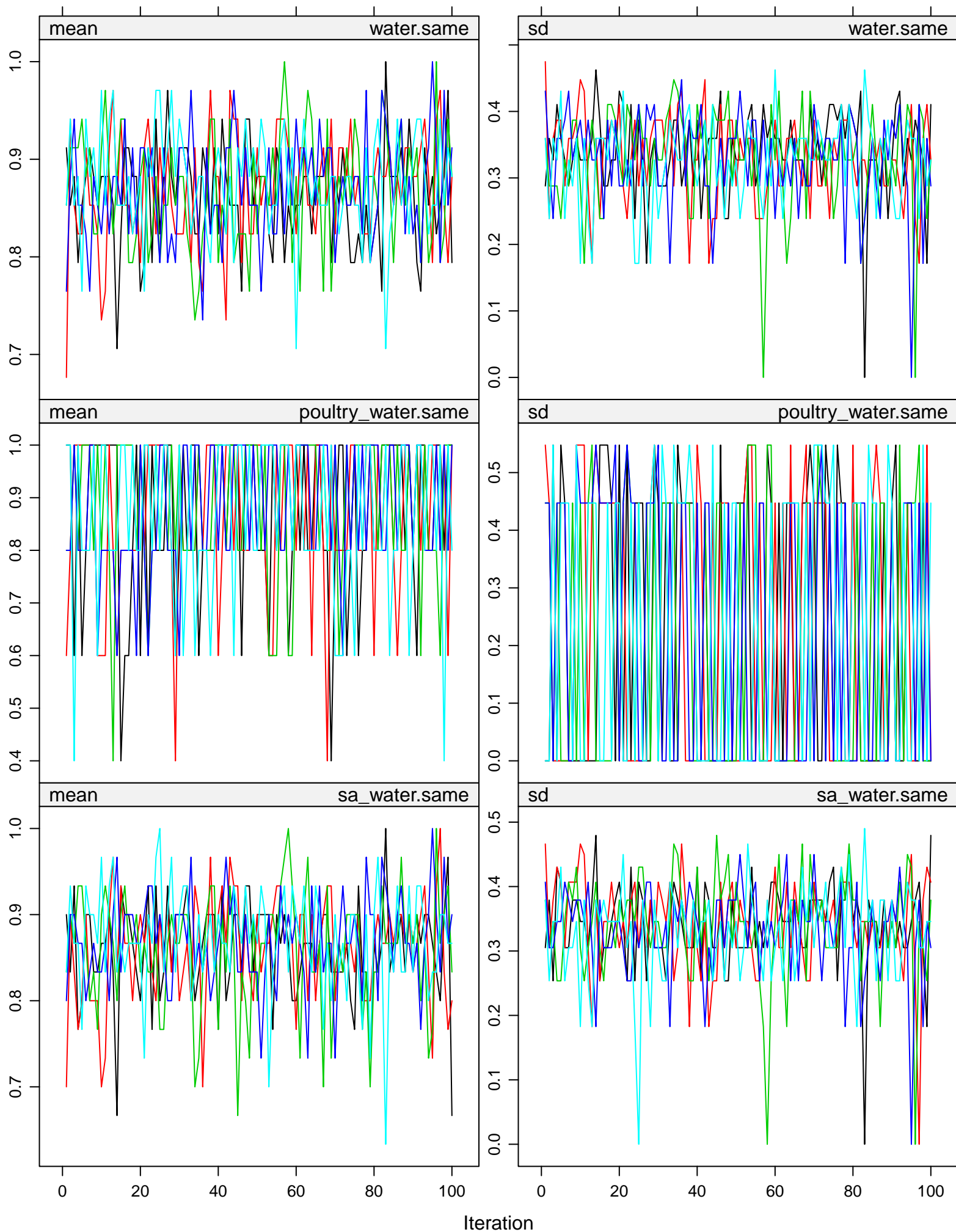

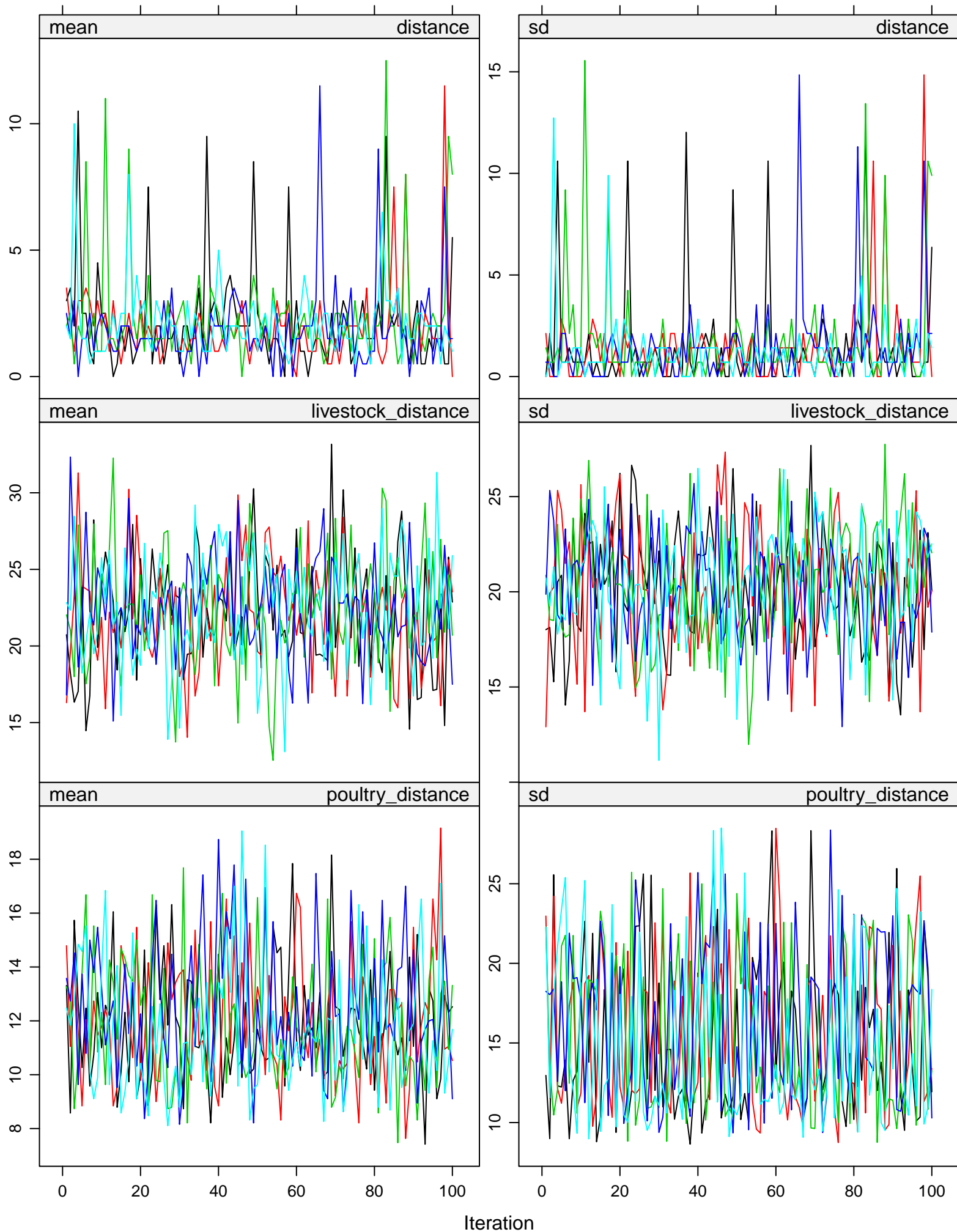

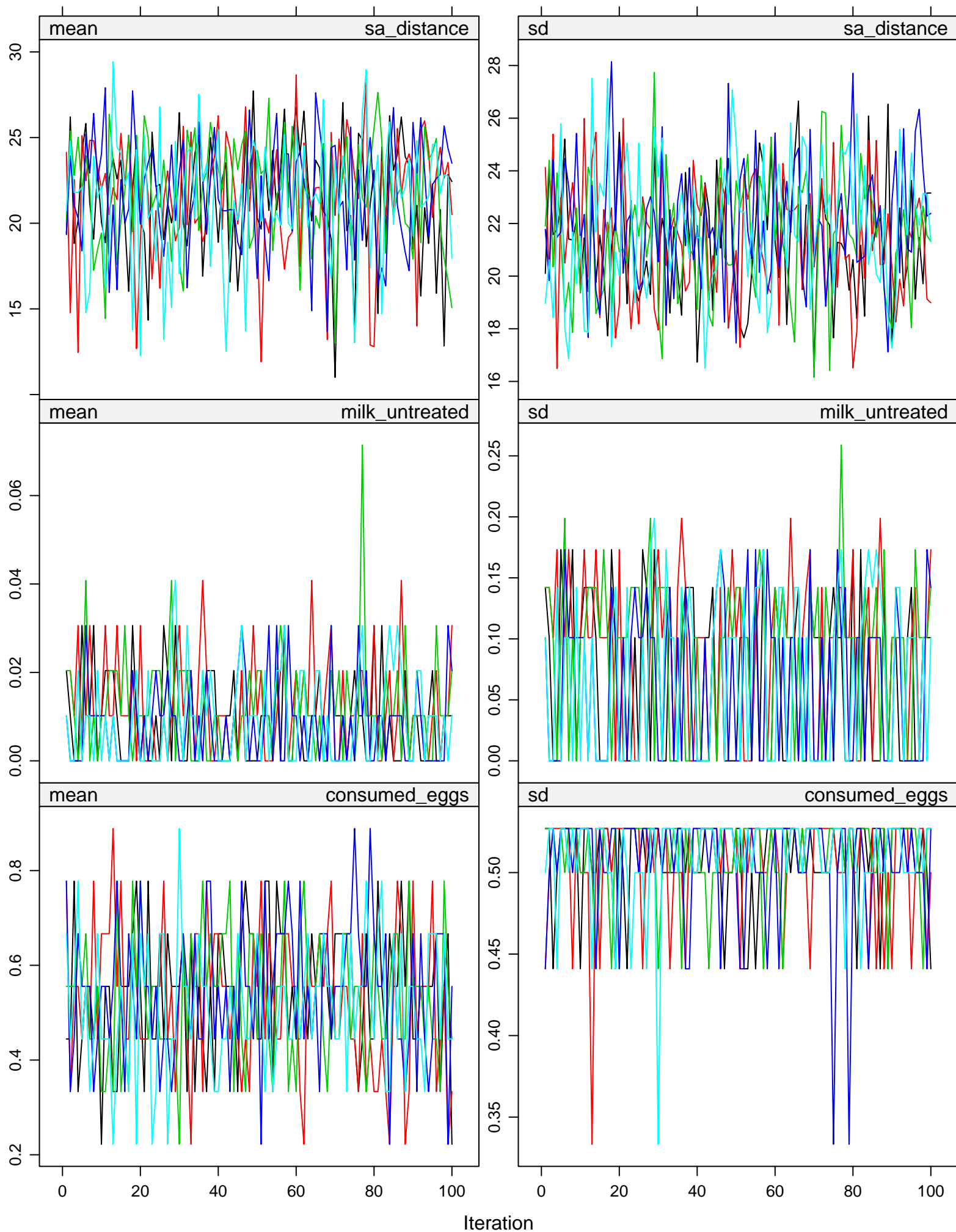

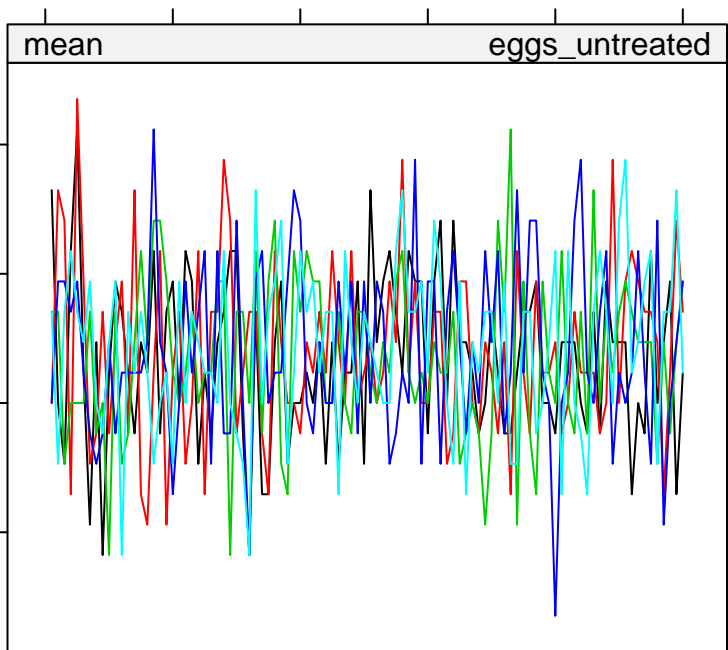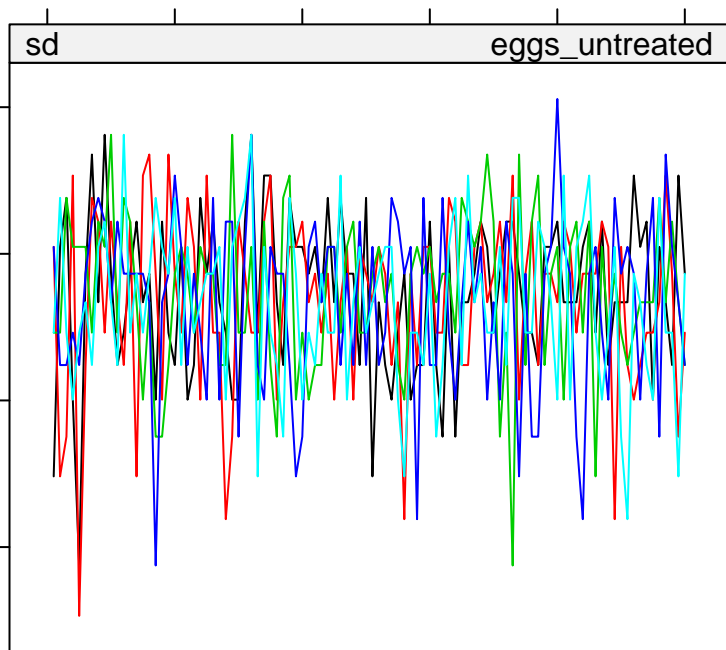

Iteration

Supplement: S1 File — Trace plots for the mean and standard deviation of each imputed variable against the iteration number. (PDF) [file pone.0215982.s004.pdf]

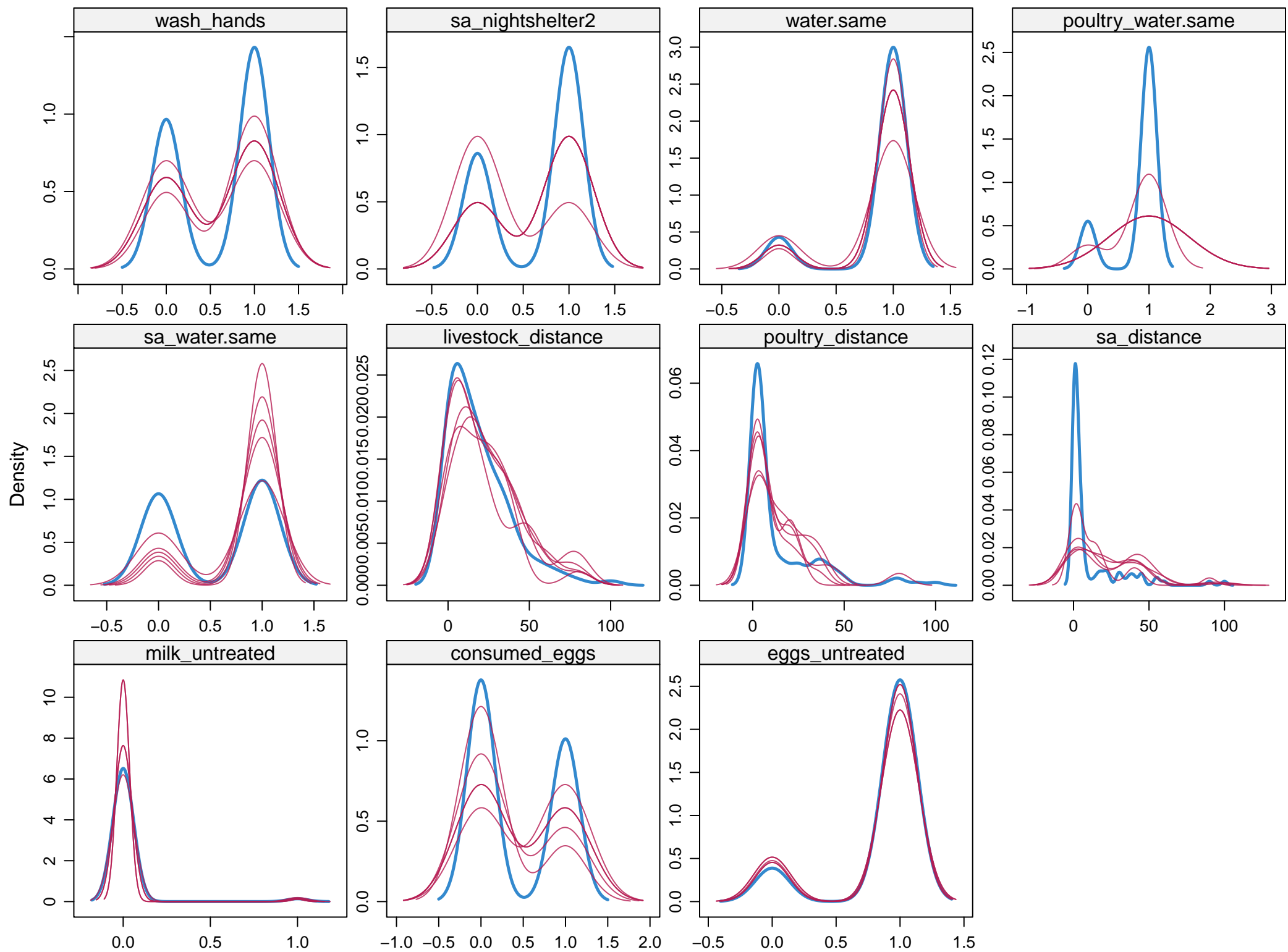

Supplement: S2 File — Density plots for each variable imputed using MICE, with observed data in blue, and imputed data (5 datasets in total) in red. (PDF) [file pone.0215982.s005.pdf]

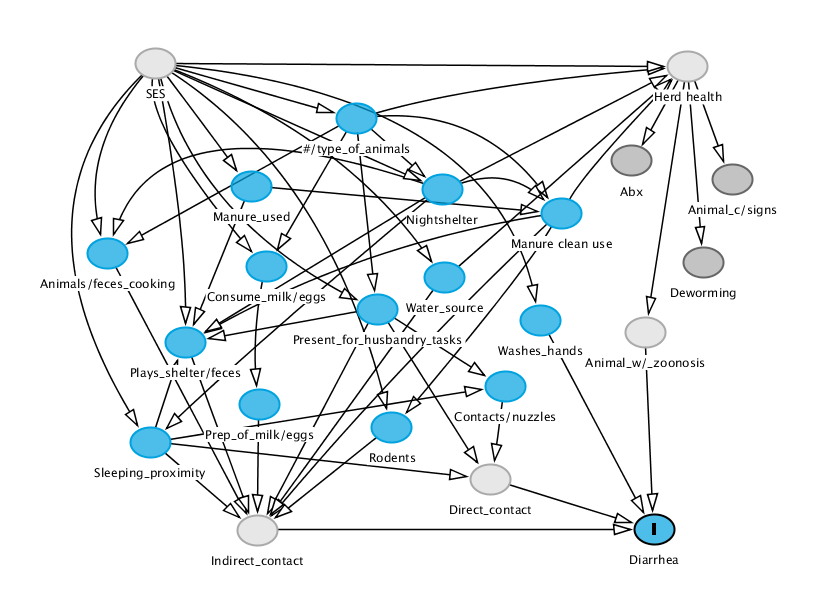

Supplement: S1 Fig — Constructed using DAGitty.net. This DAG is additionally published on DAGitty.net: Published DAG; grey nodes are unmeasured or latent variables. (TIF) [file pone.0215982.s006.tif]

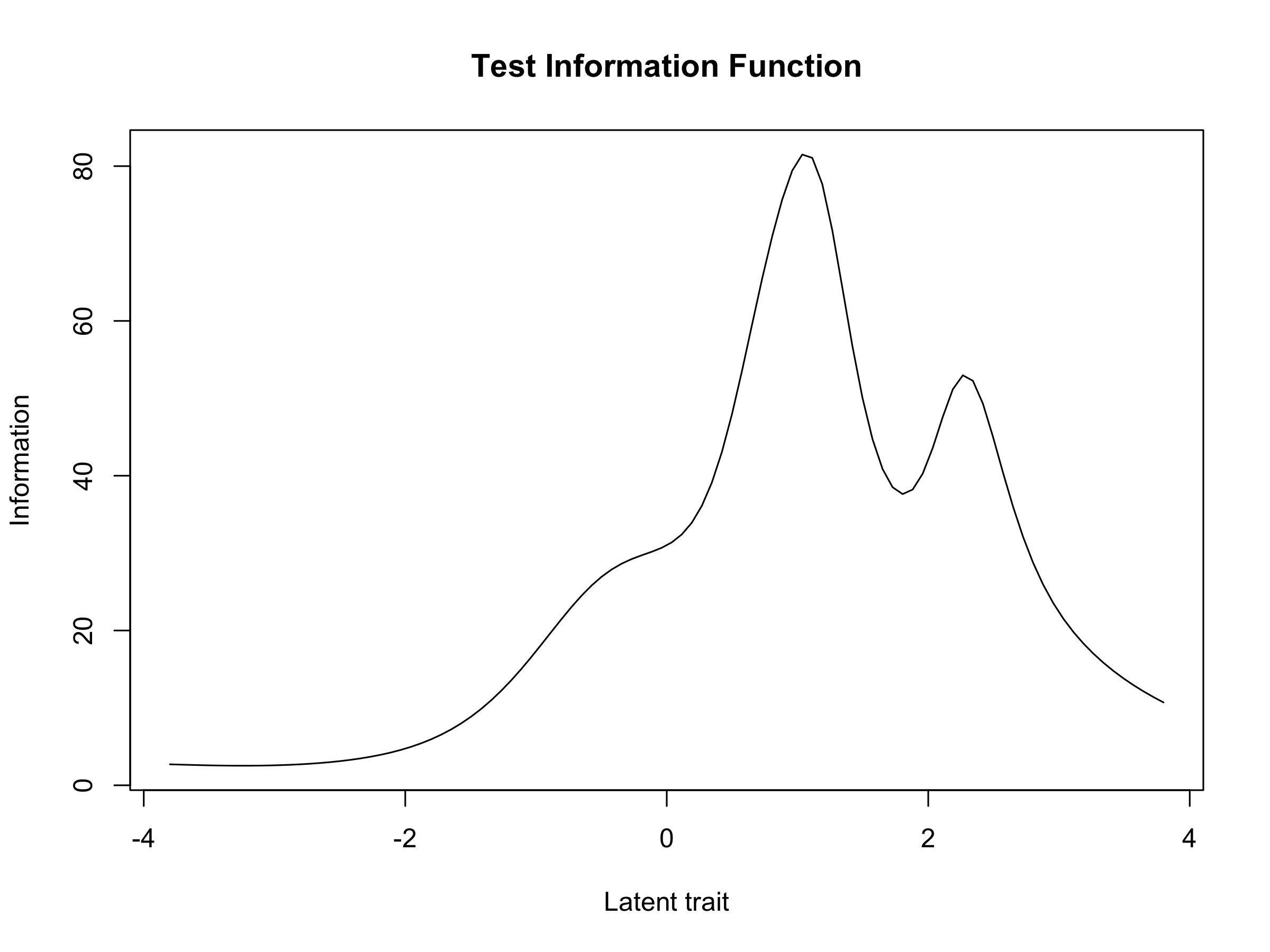

Supplement: S2 Fig — Test information function from latent variable analysis, which shows how the information contained in these items changes at different levels of the latent trait. The information function has two peaks, one large peak approximately 1 standard deviations above the mean, and a smaller peak at approximately 2.5 standard deviations above the mean. This suggests these items are good for determining average to high levels of the latent trait. (TIFF) [file pone.0215982.s007.tiff]

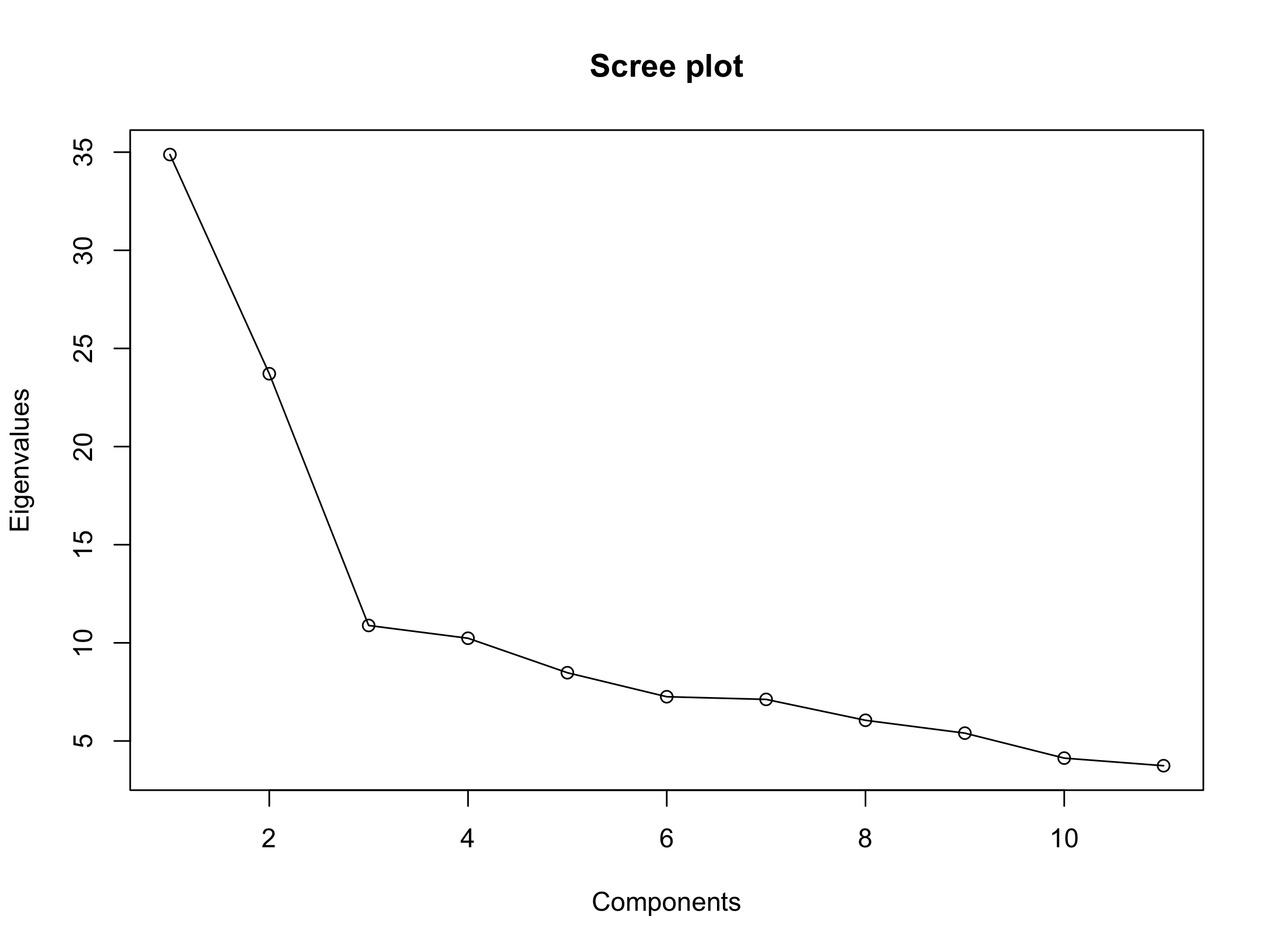

Supplement: S3 Fig — Scree plot of the first 10 eigenvalues of the tetrachoric correlation matrix to assess the dimensionality of the data. Note this correlation matrix was fit to a single imputed dataset. There is a large drop between the first and second eigenvalues, but another large drop between the second and third eigenvalues. This could be interpreted as either one or two dominant factors. (TIFF) [file pone.0215982.s008.tiff]
